# Global Genetic Heterogeneity in Adaptive Traits

**DOI:** 10.1093/molbev/msab208

**Published:** 2021-07-08

**Authors:** William Andres Lopez-Arboleda, Stephan Reinert, Magnus Nordborg, Arthur Korte

**Affiliations:** 1 Center for Computational and Theoretical Biology, University of Würzburg, Würzburg, Germany; 2 Gregor Mendel Institute, Austrian Academy of Sciences, Vienna BioCenter, Vienna, Austria

**Keywords:** evolutionary genomics, GWAS, regulation of gene expression, genetic architecture

## Abstract

Understanding the genetic architecture of complex traits is a major objective in biology. The standard approach for doing so is genome-wide association studies (GWAS), which aim to identify genetic polymorphisms responsible for variation in traits of interest. In human genetics, consistency across studies is commonly used as an indicator of reliability. However, if traits are involved in adaptation to the local environment, we do not necessarily expect reproducibility. On the contrary, results may depend on where you sample, and sampling across a wide range of environments may decrease the power of GWAS because of increased genetic heterogeneity. In this study, we examine how sampling affects GWAS in the model plant species *Arabidopsis thaliana*. We show that traits like flowering time are indeed influenced by distinct genetic effects in local populations. Furthermore, using gene expression as a molecular phenotype, we show that some genes are globally affected by shared variants, whereas others are affected by variants specific to subpopulations. Remarkably, the former are essentially all *cis*-regulated, whereas the latter are predominately affected by *trans*-acting variants. Our result illustrate that conclusions about genetic architecture can be extremely sensitive to sampling and population structure.

## Introduction

Genome-wide association studies (GWAS) have become the standard tool for analyzing the relationship between genotype and phenotype in populations. Pioneered in human genetics (Hirschhorn and Daly 2005), GWAS are now widely used in many different species to infer trait architecture and identify causal variants. Much less work has been done on comparing architectures across populations. Repetition of GWAS in different human populations or samples have mostly been used in meta-studies to improve power, although awareness is growing that genetic architecture may be different between populations (Turley et al. 2021), although difference between human populations may also reflect uncontrolled environmental differences (Barton et al. 2019; Berg et al. 2019; Sohail et al. 2019).

However, when working on traits that are likely to be involved in local adaptation, there is every reason to expect differences in the underlying genetic architecture. We expect allele frequency shifts for loci that are under selection. The magnitude of the changes will depend on the spatial or temporal scale, as well as on the strength of selection (Fraser et al. 2011; Le Corre and Kremer 2012; De Kort et al. 2015).

How does this genetic heterogeneity affect GWAS and what can we learn from it? To address these questions, we used data from the model plant species *Arabidopsis thaliana*, which occurs throughout the northern hemisphere, and has been shown to be locally adapted (Fournier-Level et al. 2011; Hancock et al. 2011; Ferrero-Serrano and Assmann 2019). Indeed, the importance of geographic scale in choosing mapping populations for GWAS has been already stressed for this species (Brachi et al. 2013).

Our general strategy was to compare the GWAS results from a global sample to various regional subsamples. We started using flowering time as a trait, since it is well studied, subject to strong selection (Flowers et al. 2009; Ågren et al. 2017) and well-understood molecularly in *A. thaliana* (Henderson and Dean 2004) and in other plant species (Weller and Ortega 2015). We also analyzed stomata size and cauline leaf number as additional phenotypes, and compared the results with simulations to establish how GWAS in subpopulations would be expected to behave under simple models. Finally, we performed GWAS on gene expression levels to investigate whether gene regulation shows evidence of local adaptation.

## Analysis

### Flowering Time Is Affected by Different Alleles in Different Populations

**Fig. 1.**
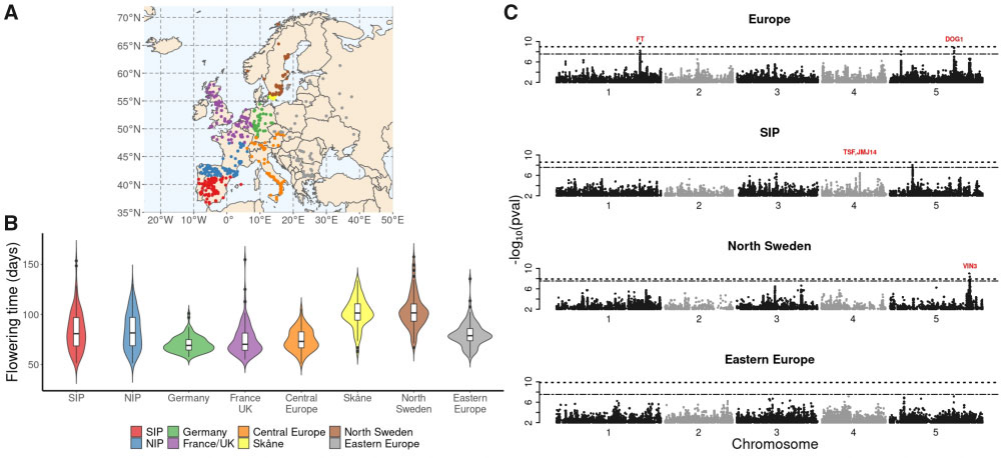
GWAS of flowering time across Europe. (*A*) Origin of the 888 European *Arabidopsis thaliana* accessions, with eight designated subpopulations in different colors. (*B*) The distribution of flowering time in the subpopulations. (*C*) Manhattan plots of GWAS results for the whole European populations and three of the eight subpopulations. Dashed and dash-dotted lines indicate Bonferroni- and permutation-based 5% significance thresholds, respectively. Candidate genes that are in close proximity to significantly associated markers are indicated in red.

We used publicly available data on flowering time, measured in growth chambers at 10 °C for over 1,000 accessions (1001 Genomes Consortium 2016). We restricted our analysis to 888 accessions from Europe and divided those into eight semiarbitrary subpopulations of approximately equal sizes using only geographic information: Southern Iberian Peninsula (SIP), Northern Iberian Peninsula (NIP), Germany, France/UK, Central Europe, Skåne (the southernmost province of Sweden), Northern Sweden (Sweden excluding Skåne), and Eastern Europe (fig. 1*A* and supplementary table S1, Supplementary Material online). All subpopulations had highly variable flowering times, with only the two Swedish ones being generally later-flowering (fig. 1*B*).

We performed GWAS on the entire European population as well as in the different subpopulations. Note that, although the subpopulations are small (n=103−119), flowering time has extremely high heritability and major polymorphisms are believed to be common (Mouradov et al. 2002). Simulations suggested that power should be sufficient to identify such polymorphisms (supplementary table S1, Supplementary Material online), and this is born out by results that pinpoint several well-known genes (fig. 1*C* and supplementary fig. S1, Supplementary Material online, table 1).

**Table 1:** Significant SNPs (chromosome:position) in the GWAS of Different Subpopulations

SNP	1:24339560	3:3458977	4:10949262	4:11016778	5:18590501	5:23100540	5:23234243
Candidate gene	*FT* ^a^		*TSF* ^b^, *JMJ14*^c^	*TSF*, *JMJ14*	*DOG1* ^d^	*CIR1* ^e^, *VIN3*^f^	*CIR1*, *VIN3*
Europe	2.4e-10 (0.44)	4.3e-03 (0.18)	4.1e-01 (0.34)	1.4e-04 (0.25)	1.7e-09 (0.20)	9.9e-10 (0.03)	1.4e-06 (0.07)
SIP	1.7e-02 (0.45)	5.1e-02 (0.47)	9.3e-01 (0.19)	2.0e-09 (0.12)	2.9e-02 (0.03)	5.2e-01 (0.01)	7.4e-01 (0.04)
NIP	1.2e-02 (0.35)	1.2e-01 (0.35)	8.2e-01 (0.32)	6.8e-02 (0.22)	1.8e-08 (0.14)	5.2e-01 (0.04)	5.8e-01 (0.10)
Germany	3.1e-02 (0.28)	9.3e-01 (0.05)	6.4e-01 (0.49)	9.4e-01 (0.28)	2.6e-02 (0.06)		2.4e-01 (0.06)
France/UK	7.0e-04 (0.41)	2.9e-01 (0.08)	6.4e-01 (0.50)	9.5e-01 (0.17)	1.4e-01 (0.05)		8.2e-01 (0.08)
Central Europe	7.4e-02 (0.46)	4.1e-08 (0.22)	1.2e-08 (0.37)	1.1e-01 (0.12)	8.2e-02 (0.05)		
Skåne	5.1e-02 (0.24)	2.1e-01 (0.12)	5.7e-01 (0.36)	8.5e-02 (0.49)	1.5e-01 (0.33)		2.7e-01 (0.01)
Northern Sweden	3.1e-01 (0.20)	9.7e-01 (0.13)	9.1e-01 (0.22)	4.2e-01 (0.37)	1.0e-01 (0.48)	9.8e-10 (0.20)	4.3e-09 (0.24)
Eastern Europe	2.6e-01 (0.44)	6.2e-01 (0.09)	7.4e-01 (0.26)	2.8e-01 (0.14)	7.4e-08 (0.08)		4.1e-01 (0.01)

Note.—Entries are “*P* value (minor allele frequency),” with genome-wide significance using a 5%-permutation-based threshold shown in red. Candidate genes were assigned to the SNPs from a list of 306 flowering time genes (Bouché et al. 2016) using 10-kb window.

a *FT* (*FLOWERING LOCUS T*, Corbesier et al. 2007).

b *TSF* (*TARGET OF FLC AND SVP1*, Yamaguchi et al. 2005).

c *JMJ14* (*JUMONJI 14*, Lu et al. 2010).

d *DOG1* (*DELAY OF GERMINATION 1*, Huo et al. 2016).

e *CIR1* (*CIRCADIAN 1*, Zhang et al. 2007).

f *VIN3* (*VERNALIZATION INSENSITIVE 3*, Sung and Amasino 2004).

Using a permutation-based threshold (Freudenthal et al. 2019), we identified genome-wide significant associations in four of the subpopulations as well as in the full European population (table 1 and supplementary tables S2 and S3, Supplementary Material online). The results differed strikingly, with only one association, near *DOG1*, showing any signs of significance in more than one subpopulation. This association was significant in NIP, almost significant in Eastern Europe, and also significant in the full population (supplementary fig. S2, Supplementary Material online). *DOG1* is an extensively studied gene involved in the regulation of seed dormancy (Huo et al. 2016; Kerdaffrec et al. 2016), but has also been identified in GWAS for flowering time (Atwell et al. 2010). Interestingly, associations of *DOG1* with flowering time have previously been observed at the global, but not local scales (Brachi et al. 2013).

Whether a causative polymorphism is detected or not depends on its effect size, its frequency, and whether it is “tagged” by a marker included in the study. The latter is a major concern when comparing human populations, because sparse SNP data are used, and patterns of linkage disequilibrium can differ greatly between populations (Martin et al. 2017). Although this explanation cannot be excluded here, it is likely to be much less important, because we are using dense SNP data from whole-genome resequencing. Compared with a standard human GWAS, we are using four times as many markers in a genome that is 25 times smaller, but in which linkage disequilibrium is roughly as extensive (Nordborg et al. 2002; 1001 Genomes Consortium 2016). Thus, even if some of the causal variants are structural (e.g., transposon–insertions), they mostly will be captured by the extensive local haplotype structure.

The other two explanations are more interesting. For polymorphisms involved in local adaptation, allele frequencies are expected to differ between geographic regions, and the *VIN3* association (5:23100540) may be an example of this. The minor allele at this locus appears to be associated with late flowering across Europe, but is too rare to be detected except in Northern Sweden (table 1 and supplementary fig. S3, Supplementary Material online).

However, we also see several examples of SNPs that are common everywhere, but show no sign of being associated with flowering time except for a single population. As noted above, differences in linkage disequilibrium with closely linked unobserved causal polymorphisms are impossible to rule out, but we think it is more likely that the difference is the broader genetic background, which could influence the effect size through epistatic interactions with other loci or via genome-wide linkage disequilibrium caused by population structure or selection (Yu et al. 2006; Vilhjálmsson and Nordborg 2013).

In our analyses, the effect of the genetic background is estimated using a mixed model, and marginal marker effects are estimated independently in a single-locus model (Kang et al. 2008). These estimates should in principle be unbiased, but there is no guarantee that this will be the case if the assumptions of the model (notably a polygenic, additive background, and normally distributed residuals) are violated.

**Fig. 2.**
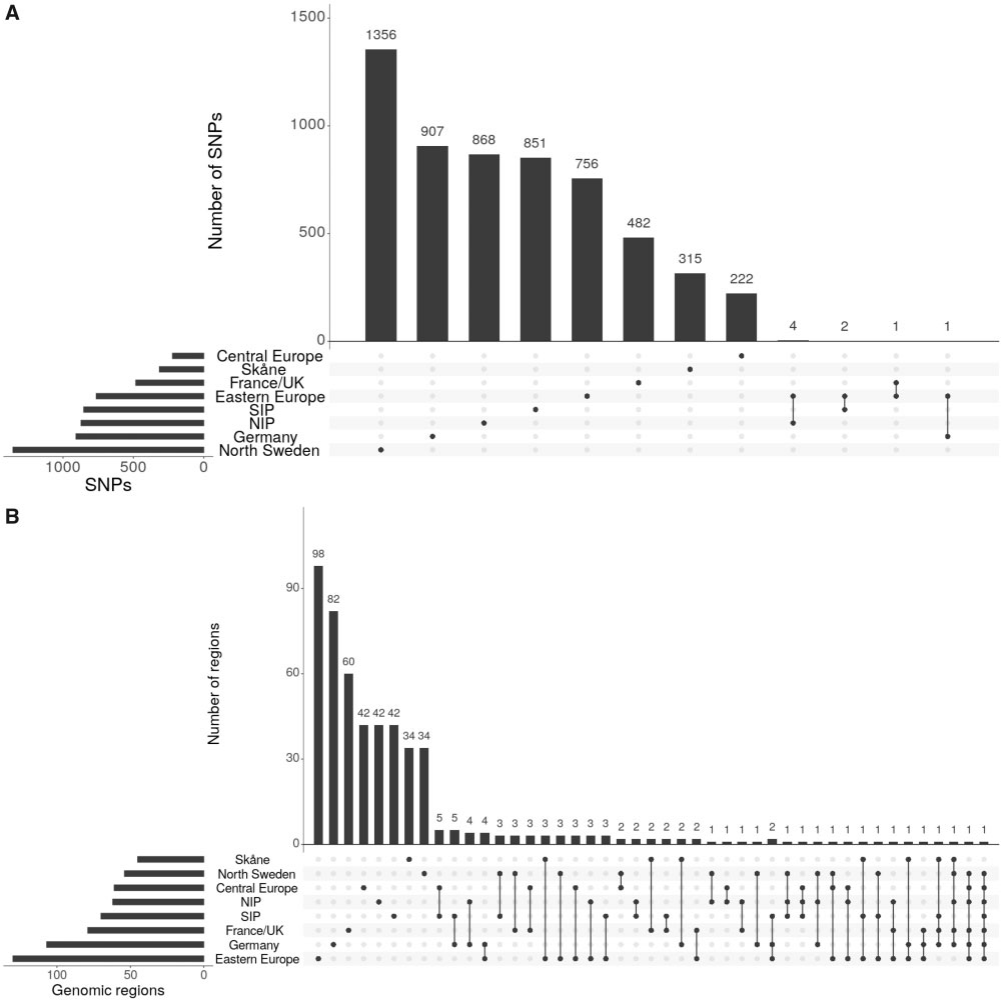
Sharing of subsignificant ($P<10^{-4}$) associations. (*A*) Histogram of the number of associated SNPs in each subpopulation and shared between subpopulations. (*B*) Histogram of the number of associated genomic regions in each subpopulation and shared between subpopulations.

To confirm that these conclusions are not limited to genome-wide significant SNPs, we next compared all SNPs with $P<10^{-4}$. In agreement with the results just presented, only eight of over 5,000 subsignificant SNPs were shared among subpopulations, and associations were never shared among more than two (fig. 2*A* and supplementary table S4, Supplementary Material online). Congruently with the notion that many subsignificant associations are real, this are far more associations than expected by chance; indeed, even the overlap is higher than expected (supplementary fig. S4*A*, Supplementary Material online).

Also notable is that shared subsignificant associations are clearly clustered in genomic regions that tend to be common between subpopulations (fig. 2*B*, see Materials and Methods for details). Significantly fewer shared regions are detected in the simulations (supplementary figs. S4*B* and S5, Supplementary Material online). Although regions shared among multiple subpopulations are located in close proximity to known flowering time genes (supplementary table S5 and file S1, Supplementary Material online), no significant enrichment had been observed.

There are two possible explanations why different SNPs in the same genomic region could be associated. The first is that the causal polymorphisms are absent from the data, and that different SNPs “tag” the (shared) causal polymorphisms in the different subpopulations. As noted above, given the high SNP density used, we do not believe this is a general explanation. More likely is extensive allelic heterogeneity, a phenomenon consistent with local adaptation, and well-demonstrated in *A. thaliana* (Atwell et al. 2010; Li et al. 2014; Kerdaffrec et al. 2016; Zhang and Jiménez-Gómez 2020).

To further investigate the putative heterogeneity, we estimated the polygenic overlap between subpopulations using a method that estimates the correlation of marker effects across different samples based on GWAS summary statistics without trying to detect significant associations and thus potentially biasing the results (Frei et al. 2019). Although this method predicts the existence of shared genetic variants, the correlation of the respective effect sizes varies across subpopulations and the overall correlations of all marker effects were very low in all comparisons. As a contrast, the marker-effect correlation between flowering time at 10 and 16 °C (FT16) was quite high (supplementary table S6 and fig. S6, Supplementary Material online). This again supports the notion of different architectures in different subpopulations.

### Simulations Suggest That Local Genetic Architecture Is Detectable

Flowering time is the quintessential locally adaptive trait. It is difficult to know how unusual it is, because few traits have been measured in different populations in wild species. Even in *A. thaliana*, few relevant data sets exist. The most relevant phenotypes we were able to find were stomata size and cauline leaf number, measured in 131 accessions from Sweden and 109 from the Iberian Peninsula (supplementary fig. S7, Supplementary Material online). However, the analysis was uninformative, as no genome-wide significant associations were identified (supplementary fig. S8, Supplementary Material online), and no overlap was found for subsignificant associations either (supplementary fig. S9, Supplementary Material online). Indeed, despite both phenotypes having high heritabilities (27–85%; supplementary table S7, Supplementary Material online), the joint *P* value distribution was indistinguishable from noise, suggesting that GWAS is underpowered to detect causal alleles for these phenotypes. A potential explanation could be that both phenotypes are highly polygenic, and major alleles do not exist. Alternatively, these samples have low power because of population structure: this is supported by the fact that we do not find significant association for flowering time in these samples either.

To gain insight into the power to detect causal alleles in these samples, we turned to simulations. Briefly, we simulated phenotypes using the 131 Swedish and 109 Iberian accessions. A single randomly picked polymorphism was assumed to explain a fixed percentage of the phenotypic variation, in either the Swedish, the Iberian, or the merged population (see Materials and Methods for details). We calculated the power to identify the causal polymorphism as well as the number of false positive associations using a Bonferroni threshold and a thousand simulations for each scenario (supplementary tables S8 and S9 and fig. S10, Supplementary Material online). In summary, the simulations suggested that GWAS in our small populations have sufficient power to identify major alleles and population-specific effects—supporting our claim of local adaptation for flowering time, and also that major alleles for the control of stomata size and cauline leaf number do not exist, at least not in these subpopulations.

### Gene Expression Can Be Regulated Globally or Locally

Finally, we carried out GWAS on gene expression data for a large sample of world-wide accessions (Kawakatsu et al. 2016). It seemed a priori likely that at least some genes are under local selection. For comparison with the results above, and because reasonably dense local samples were available, we focused on 91 accessions from the Iberian Peninsula (IP) and 74 accessions from Scandinavia (termed SW, as nearly all accessions are from Sweden). We analyzed each subpopulation separately, as well as the merged set of 165 accessions. Because of the small sample sizes, we only considered genes with high estimated heritability and for which simulations indicate sufficient power in all three populations (see Materials and Methods). These criteria led to the retention of 2,237 genes, 9% of the total (supplementary table S10, Supplementary Material online). We also excluded genes where inflated significance levels where observed: this further reduced the number of genes to 1,982.

**Fig. 3.**
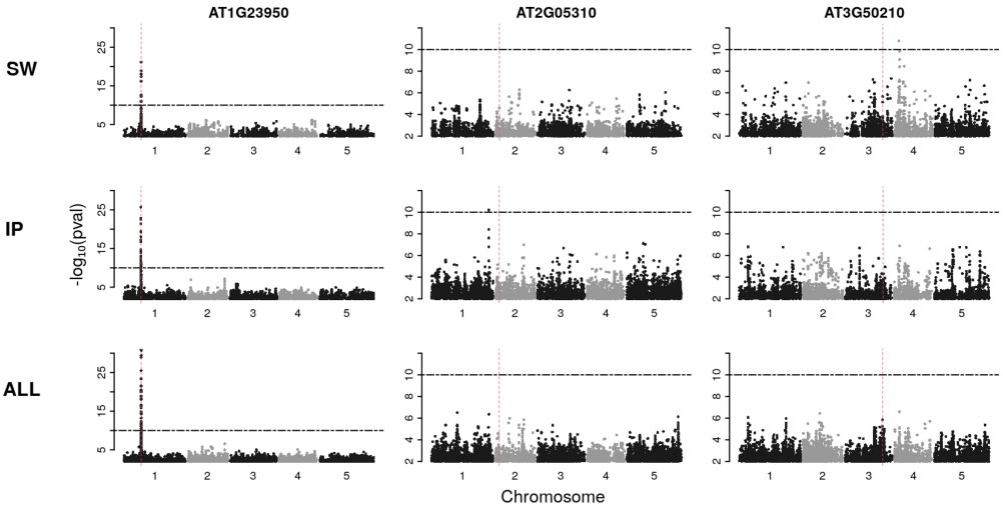
Manhattan plots from GWAS on expression levels for three different genes. The columns show the results from genes representing different scenarios. The rows display the GWAS results of the analysis in the two subpopulations (SW and IP, respectively), or in the merged population (ALL). Horizontal dash-dotted lines indicate the significance threshold of $P<10^{-10}$. Vertical dashed lines show the position of the gene whose expression is being used as a molecular phenotype.

Perhaps not surprisingly, 780 (39%) of these filtered genes revealed a genome-wide significant association (using a multiple-testing corrected threshold of $P<10^{-10}$) in at least one of the two subpopulations (typical results are shown in fig. 3). These genes were divided according to the pattern of associations within and between subpopulations, with the intent to identify those with clear evidence for global versus local genetic architecture (see supplementary fig. S11, Supplementary Material online and Materials and Methods, for details).

We found clear examples of both. Of the 780 genes with a significant association, 110 (14%) were significantly associated with the same SNP in both subpopulations (shared architecture), 25 (3%) were significantly associated with different SNPs in the same 50-kb genomic region in the both subpopulation (presumably allelic heterogeneity), 92 (12%) were significantly associated with different SNPs at distinct genetic regions in the two subpopulations (genetic heterogeneity), and 182 (23%) appeared to show an specific association in one subpopulation only (also genetic heterogeneity). The remaining are more ambiguous (supplementary fig. S11, Supplementary Material online).

**Fig. 4.**
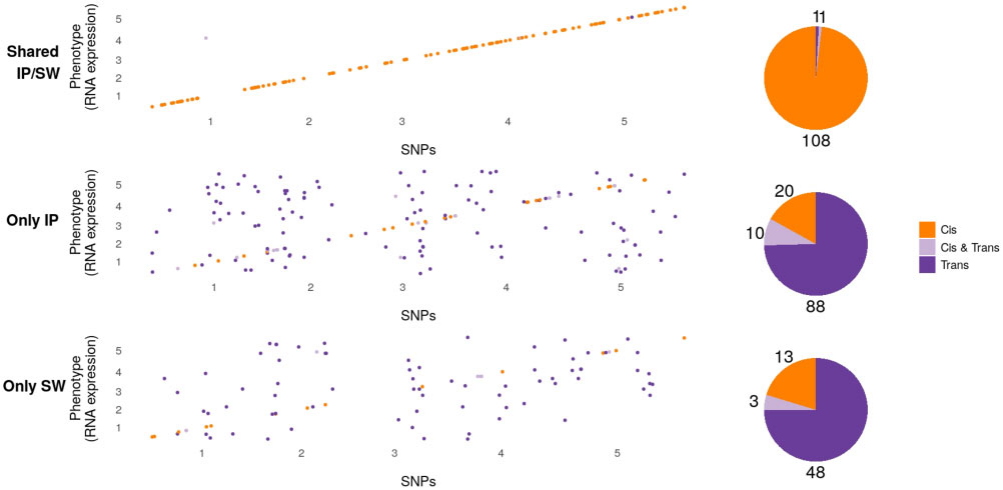
Summary of the difference between shared and nonshared GWAS results for expression data. The top panel shows associations that are shared between the two subpopulations, whereas the bottom panels show associations that are specific to one subpopulation. The plots show the chromosomal location of the genes whose expression is mapped on the *x* axis, and the chromosomal position of significantly associated SNPs on the *y* axis. Associations in *cis* are shown in orange, whereas *trans*-associations are shown in purple. The pie charts show the number of genes in each category.

Unexpectedly, we also found an extremely strong pattern of *cis-* versus *trans-*regulation. Of the 110 genes with shared association between subpopulations, 99% were *cis*-regulated, whereas the opposite was true for genes with different regulation in the subpopulations. Here, 75% of the 182 genes that appeared to show a specific association in one subpopulation only were *trans*-regulated (fig. 4 and supplementary fig. S11, Supplementary Material online).

To confirm that these results reflect real differences between the subpopulations, we generated two random populations of the same size by permuting the subpopulation labels. As expected, this recovered the shared *cis*-associations (157 genes showed shared associations, of which 94% are in *cis*, supplementary figs. S14 and S15, Supplementary Material online). Nonshared associations were still mostly in *trans*, but there are less than half as many clearly subpopulation specific ones (supplementary figs. S14–S16, Supplementary Material online). This suggests that a substantial fraction of the specific associations found in the Scandinavian and Iberian populations are real. Further supporting this, only five genes showed a pattern of allelic heterogeneity in the analysis of the random subpopulations.

A GO-enrichment analysis found a significant enrichment for “ADP binding” among genes displaying a global architecture, whereas no significant enrichment for those with local associations was found. More anecdotally, the group of genes with shared variants contains many genes linked to primary metabolic pathways, as well as genes like *RPS5* (*RESISTANT TO P. SYRINGAE 5*), which is linked to bacterial and downy mildew resistance (Warren et al. 1998), and which is likely to be under global balancing selection (Tian et al. 2002). The set of genes with local architecture contains genes related to flowering time regulation, like *AGL-20* (*AGAMOUS-LIKE 20*; Lee 2000), and stress response, like *RCAR5/PYL11* (*REGULATORY COMPONENT OF ABA RECEPTOR 5/PYRABACTIN RESISTANCE-LIKE 11*; Lim and Lee 2020) and *HDA9* (*HISTONE DEACETYLASE 9*; Zheng et al. 2016).

## Discussion

It has been clear for over a decade that GWAS in plants often produce results that are strikingly different from those typically seen in humans. Major associations explaining substantial fractions of the phenotypic variance are common, likely because this variance is adaptive, and the allelic variants are maintained by selection (Atwell et al. 2010; Huang et al. 2010). A prediction from this is that we do not necessarily expect GWAS results to replicate between populations, because many traits are likely to be involved in local adaptation (Brachi et al. 2013). Here, we use a simple analysis to show that this is very much the case for flowering time, a trait known to be important for local adaptation. We then show that the same is true for expression variation at many genes, and discover a striking pattern in that regulatory variants that are shared between populations are almost all in *cis*, whereas those that are not (and are thus suggestive of local adaption) are predominantly in *trans*.

That local adaptation would frequently involve *trans*-regulation is perhaps not surprising, as it seems likely that such adaptation generally involves expression changes at large numbers of loci. Many studies assume that polygenic adaptive traits are influenced by multiple loci with small effects, with contributions from only a few loci with larger effects (Savolainen et al. 2013). This is surely easier to achieve using variation at upstream regulatory loci. Additionally, our observation is also consistent with findings from *A. thaliana* that genotype-by-environment interactions in gene expression are mostly due to *trans*-acting variants (Clauw et al. 2016), and that, analogously, tissue-specific expression variation in humans also tends to be due to *trans*-acting variation (GTEx Consortium 2017).

The role of *cis*-regulatory variation under this scenario is less clear. Our finding that regulatory variants shared across populations are generally *cis-*acting is again reminiscent of the results of Clauw *et al.* (2016), who found that *cis*-regulatory variants had similar effect in drought and nondrought conditions. It should be noted, however, that we also found genes with allelic heterogeneity in their *cis*-regulation. This pattern is not consistent with neutral evolution, but with selection driving the diversification of *cis*-regulatory regions for genes that are linked to local adaptation.

More generally, it is important to emphasize that we have no experimental data on fitness, merely an observation of striking differences in the architecture of expression variation between subpopulations that intersect differences in *cis-* versus *trans-*regulation. Our data are consistent with a very simple model of local adaptation via *trans-*regulation, but this is surely not the only interpretation. That gene expression may be important for local adaptation has been suggested by many authors, and the role of *cis-* versus *trans-*regulation has been debated (Fraser 2013; Schaefke et al. 2013; Mähler et al. 2017; Josephs et al. 2020; He et al. 2021). The pattern we report demands an explanation, and investigating this further in proper experiments (ideally in the field), including other species, would surely be of great interest.

Our findings also have important implications for the design and interpretation of GWAS. As part of the “1001 Arabidopsis Genomes Consortium,” we have often been asked “Which subset of accessions should I use?.” This paper shows that there is no simple answer. Clearly, what you find depends on where you look, and the optimal design depends on the question as well as on the phenotype. Environmental and ecological factors vary across different scales. A global sample may not have the power to detect locally important allelic variation, and a local sample may not even contain globally important variants. Depending on the nature of reality, you will always miss some part of the picture, and if you are not aware of this, you may draw the wrong conclusions. For example, the relatively importance of *cis*- versus *trans*-regulation has been much debated (reviewed in Signor and Nuzhdin 2018), but this paper show that the answer may depend on how you sample. In conclusion, GWAS works, but should be used with caution.

## Materials and Methods

### Plant Material and Phenotypic Data

The phenotypic data used in this study were obtained from the *A. thaliana* phenotype repository AraPheno (Seren et al. 2017). The genotypic data were obtained from the 1001 Genomes Consortium (1001 Genomes Consortium 2016). Phenotypic traits used in the present study include flowering time at 10 °C (FT10, https://arapheno.1001genomes.org/phenotype/261/), flowering time at 16 °C (FT16, https://arapheno.1001genomes.org/phenotype/262/), stomata size (ST, https://arapheno.1001genomes.org/phenotype/750/), and cauline leaf number (CL, https://arapheno.1001genomes.org/phenotype/705/). AraPheno stores 1,163 world-wide *A. thaliana* accessions. We split the 888 European accessions into eight subpopulations of approximately equal sizes (103–119 accessions) (fig. 1 and supplementary table S1, Supplementary Material online). For ST and CL, the total number of accessions used in our analyses was 240. For both traits, the initial group of 240 accessions was split into two geographic subpopulations, one containing 109 Iberian accessions and the other 131 Swedish accessions. In addition to these traits, we used expression data (Kawakatsu et al. 2016) for 24,175 genes measured in 727 different accessions, available via AraPheno (https://arapheno.1001genomes.org/study/52/). We selected the 665 accessions with full genome sequencing data, and created two subpopulations roughly matching the cauline leaf and stomata size data. The “Swedish” subpopulation contains 70 accessions from Sweden, 2 accessions from Denmark, and 2 accessions from Norway, whereas the second subpopulation from the Iberian Peninsula contains 83 accessions from Spain and 8 accessions from Portugal. The RNA-seq data have been generated in two distinct batches (Yoav Voichek, personal communication), but accessions from both subpopulations were predominantly present in the second batch, minimizing the risk of batch effects in the analyses.

### Genome-Wide Association Studies

GWAS was performed using a liner mixed model to account for population structure. We used a custom R script (available at https://github.com/arthurkorte/GWAS) implementing a fast approximation of the described in Kang et al. (2010). Significance thresholds were defined using both Bonferroni- and permutation-based thresholds. The Bonferroni threshold was calculated by dividing the significance level (*α *= 0.05) by the number of SNPs with minor allele count greater five in each GWAS run. Permutation-based thresholds were derived from running 100 linear mixed models per phenotype with a random reordering of the phenotypic values (Freudenthal et al. 2019).

### Candidate Gene Enrichment

To look for an enrichment of a priori candidate genes, the regions identified as significantly associated with flowering time were been cross-referenced with a list of 306 known flowering time genes (Bouché et al. 2016). All genes within 10 kb of an associated regions were considered. This analysis was conducted with the 74 regions that were associated with flowering time in at least two subpopulations. Twenty-two of these regions overlapped with known flowering time genes. Permutation analysis by resampling random regions of the same size across the genome, showed that there is no significant enrichment of candidate genes. Neither changing the window size, nor restricting the analysis to regions that are shared in three or more subpopulation affected this conclusion.

### Simulations

In order to simulate data that mimic local and global effects, we use the same subpopulations used for the stomata size and cauline leaf GWAS. We simulated three scenarios:

1. A single marker explaining *x* % of the variance in the full population of 240 accessions;
2. A single marker explaining *x* % of the variance only in the 109 IP accessions, and;
3. A single marker explaining *x* % of the variance only in the 131 Swedish accessions.

In each scenario, the causal marker was chosen randomly from all markers with a minor allele count greater five and set to explain 20%, 15%, 10%, and 5% of the phenotypic variance, respectively. To mimic population structure, 1,000 random markers were additionally assigned random small effects that are zero-centered; 1,000 simulated phenotypes were generated for each setting, resulting in a total of 12,000 simulated phenotypes. All simulated data were generated using a custom R script (https://github.com/arthurkorte/GWAS). When the simulated causative marker explained 20% of the phenotypic variation, GWAS performed using all accessions resulted in the detection of this causative marker in 96.4% of the cases, albeit at a high false discovery rate (FDR) of 18.9%. Here, we consider an association as false, if it is more then 100 kb apart from the simulated causal marker. This high FDR dropped dramatically when a more stringent threshold of $P<10^{-9}$ or $P<10^{-10}$ was applied. Even with this more stringent threshold, a power of 87.6% and 79.4% was reported, whereas the FDR dropped to 8.4% and 4.8%, respectively. We observed a reduced power in GWAS when using the two different subpopulations (24.6% in IP and 39% in SW). The reduced detection rate of the marker in IP and SW is caused by a reduced power due to the smaller population size. If the simulations mimic a scenario of a marker having a local effect only, the respective marker was exclusively detected in the respective local subpopulation (42% in SW and 27.4% in IP) and—with a reduced power—in the analyses using all accessions (6.5% and 27%, respectively). Representative GWAS results of the simulated phenotypes are presented in supplementary figure S10, Supplementary Material online. The analyses of simulations with a reduced effect size of the causative marker led to similar results, albeit at a reduced power (supplementary table S9, Supplementary Material online).

### Polygenic Overlap

First, we estimated the polygenic overlap among all subpopulations by comparing lists of significant SNPs. Since the comparison of significant SNPs between subsets showed no shared signals, we set a less stringent *P* value threshold ($P<10^{-4}$) and generated a new list of SNPs for comparing subpopulations. Additionally, we looked at shared significant genomic regions. For this, we summarized all SNPs ($P<10^{-4}$) with either $r^{2}>0.9$ or located within a 10-kb window for each subpopulation and compared significant genomic regions. The same procedure has been performed for the respective GWAS results of the subpopulations, as well as with GWAS results from permutations within the respective subpopulation to compare the overlap to the expected overlap in a scenario where no causal markers are present.

Next, we estimated the polygenic overlap using the statistical tool *MiXeR* (Frei et al. 2019), which overcomes the intrinsic problem of detecting the exact location of shared causal variants. In short, a summary table containing SNP information, genomic location, beta estimates, and *z*-scores for each subpopulation was created and used to estimate the proportion of shared causal SNPs between subsets based on their beta and *z*-score distributions.

### RNA Expression Data

The available RNA expression data contain transcription values for 24,175 genes. Before performing GWAS on the RNA expression data, we removed TEs and genes that are encoded by the organelle genomes, leaving 23,021 nuclear genes for further analyses. Next, we selected genes where the pseudoheritability estimate was above 0.5 and a statistical power analysis estimated that the power in GWAS was greater than 0.9 (using the method of Wang and Xu [2019]). Heritability was estimated for all genes using the above mentioned implementation of the mixed model. The power of each data set for GWAS was calculated using the *pwr.p.test* function implemented in the R package *pwr* (R Development Core Team 2008). This filtering led to a set of 2,237 genes for which GWAS was performed in both subpopulations (IP and SW), as well as in the combined population (ALL). We only considered markers with a minor allele count of more than five in the respective subpopulation. Given the amount of tests we performed, we used a very stringent multiple-testing threshold of $P<10^{-10}$ to term an association as significant, but similar results have been reproduced with threshold ranging from $P<10^{-8}$ to $P<10^{-12}$. Significant associations were grouped into regions, if they occur within 50 kb of each other. A summary of the number of associated markers and regions for all analyzed genes as well as summary statistics are attached in supplementary file S2, Supplementary Material online. Genes showing inflated GWAS results (which quite often co-occurs with a nonnormal distribution of the expression values), have been filtered out, if the number of associated genomic region was greater then three in either the IP or SW subpopulation. This procedure left us with a set of 1,982 genes. GWAS results from these selected genes were analyzed in more detail and the complete workflow of the analysis is displayed in supplementary figure S11, Supplementary Material online. We identified 227 genes displaying an association in both the IP and SW subpopulation. For 135 of them, the same genomic region was associated in both subpopulations, whereas for 92 genes, different genomic regions have been associated in the two subpopulations (supplementary file S3, Supplementary Material online). To prevent genes from being assigned as locally regulated in both subpopulations, those genes have not been considered as genes displaying a local regulation. Still, these genes show the same pattern of *cis*- versus *trans*-regulation observed for genes with a specific local association. Genes, where the same genomic region was associated, were defined as genes having a global genetic regulation, if the same significant marker in both subpopulations was associated (110, supplementary file S4, Supplementary Material online), whereas genes where the same region but different markers are associated in the subpopulations (25, supplementary file S5, Supplementary Material online), were classified as genes showing potential allelic heterogeneity in their regulation. Next, genes that show an association only in one and not the other subpopulation were defined as genes that are under distinct local regulation. This led to the identification of 377 genes displaying an association only in IP and 176 genes displaying an association only in SW. Now, we filtered for genes, where the respective *P* value was lower in the analysis of the respective subpopulation compared with the results of the combined population, as we argue that a true local association should be more significantly associated in the respective local subpopulation. Additionally, we also excluded genes, where different regions have been associated in the analysis of the combined population compared with the analysis of respective local subpopulation, to generate a high confidence list of genes with a distinct regulation in only one subpopulation. This procedure led to a set of 118 genes displaying a specific local association only in the Iberian subpopulation (supplementary file S6, Supplementary Material online) and 64 genes displaying a specific local association only for the Scandinavian subpopulation (supplementary file S7, Supplementary Material online). For significant associations in these three groups of genes, having the same association in both subpopulations, a specific local association only in IP or a specific local association only in SW, we verified, if the respective associated SNPs were in *cis*, aka the same genomic region where the gene is located, or in *trans*. Here, we defined a *cis*-association, by a maximum distance of the associated markers to the respective gene of 100 kb. As a control, we also performed the same analysis described above with two random, nonlocal population of 91 and 74 accessions, respectively. These random populations have been sampled from the merged population of 165 accessions. The respective workflow and numbers are presented in supplementary figure S15, Supplementary Material online. Note, that we started out with the same set of 2,237 genes used previously, but here the removal of genes showing inflated results, led to a set of 2,087 genes included in the analysis.

### GO-Enrichment Analysis

The different lists of genes showing either globally the same regulation or a specific local architecture in one of the subpopulations where used for a GO-enrichment analysis. The analysis was performed using Gorilla (Eden et al. 2009) comparing two unranked lists of genes. Here, the respective gene lists where compared with a background list containing all 1,982 genes for which expression-based GWAS was performed.

## Supplementary Material

Supplementary data are available at *Molecular Biology and Evolution* online.

## Supplementary Material

msab208_Supplementary_Data
