# Supplementary material for: Global Genetic Heterogeneity in Adaptive Traits
Source: Mol Biol Evol. 2021 Jul 8;38(11):4822–31. doi: 10.1093/molbev/msab208 (PMC8557469; doi:10.1093/molbev/msab208)
Supplement: msab208_Supplementary_Data [file msab208_supplementary_data.zip › Global_genetic_heterogeneity_in_adaptive_traits_revision_supplement.pdf]

1 Supplementary Figures

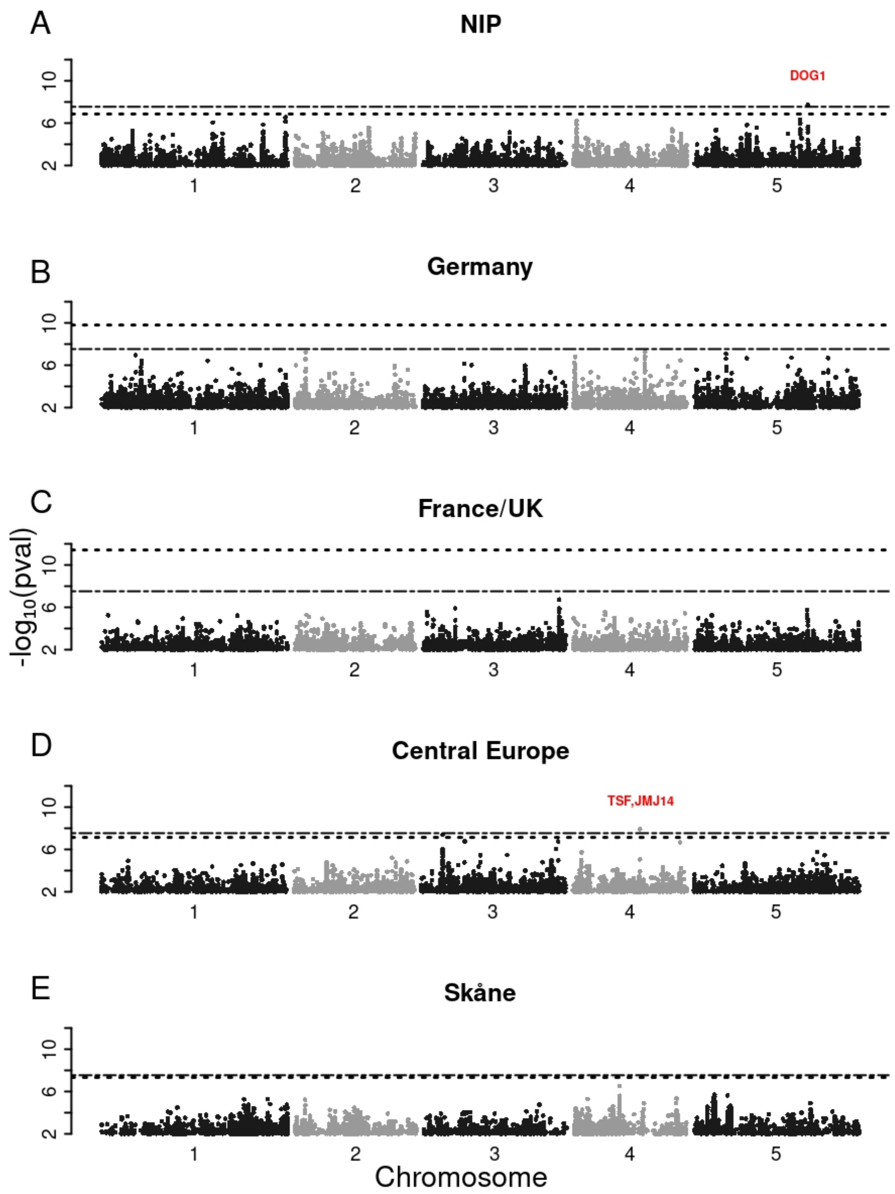

Supplementary Fig. 1: Manhattan plot for the GWAS results of flowering time in five different subpopulations. Dashed lines and dash-dotted lines indicate 5% permutation-based and Bonferroni threshold, respectively.

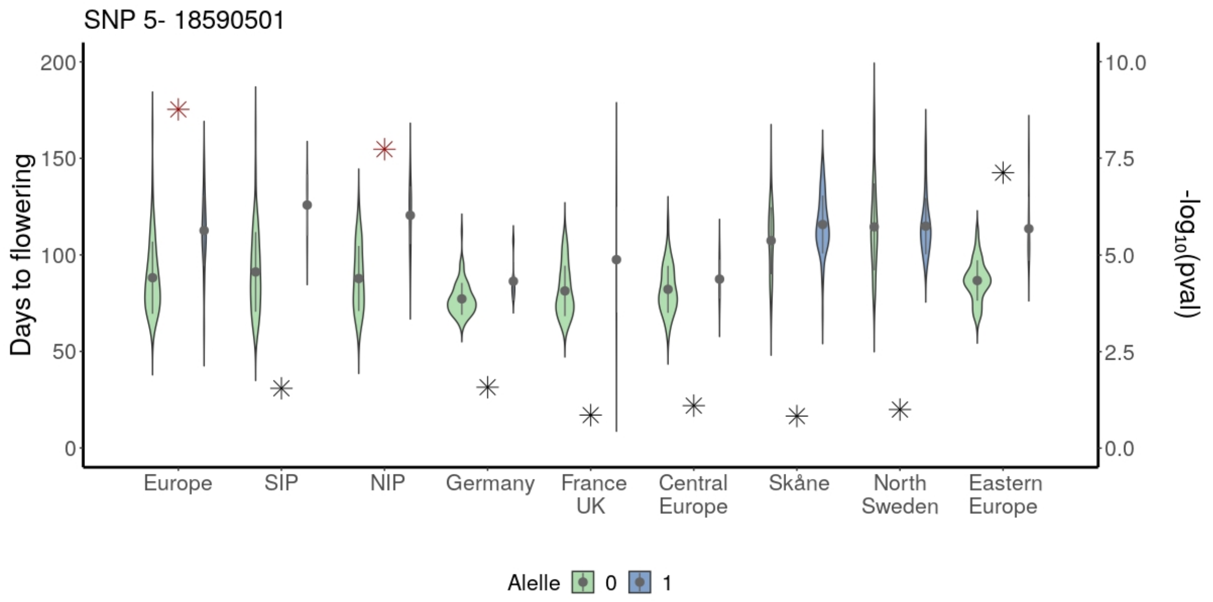

Supplementary Fig. 2: Violin plots comparing flowering time between accessions carrying reference and alternative allele for the SNP 5:18590501. Stars represent the -$\log_{10}$ of the p-value and a red colored star indicates a significant association.

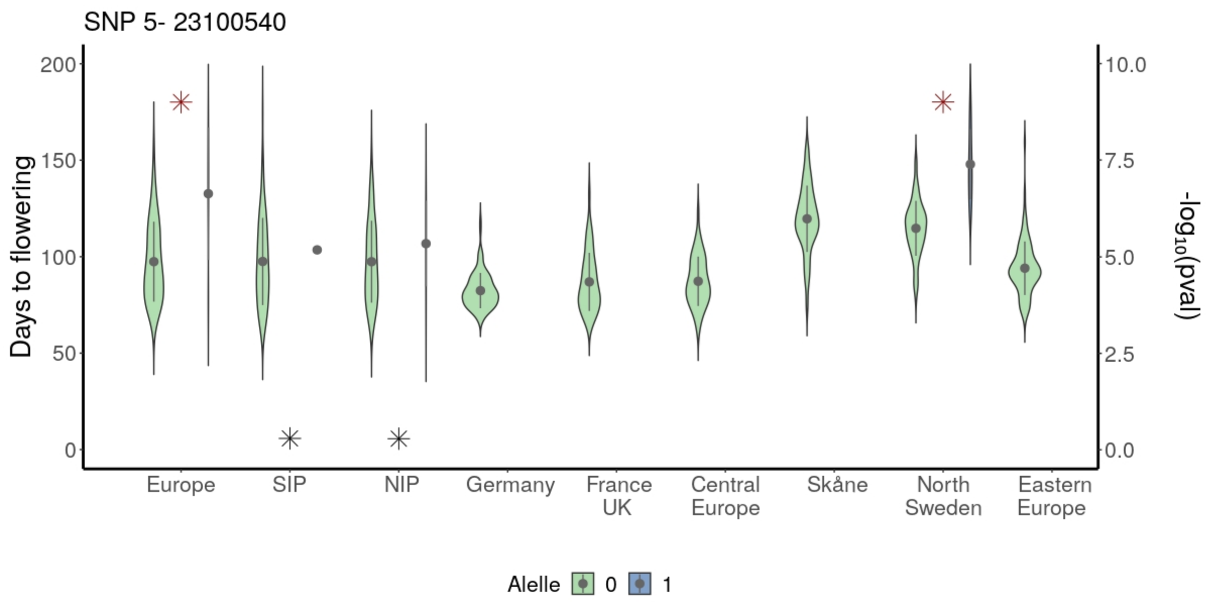

Supplementary Fig. 3: Violin plots comparing flowering time between accessions carrying the reference or alternative allele for SNP 5:23100540. Stars represent the -$\log_{10}$ of the p-value and a red colored star indicates a significant association.

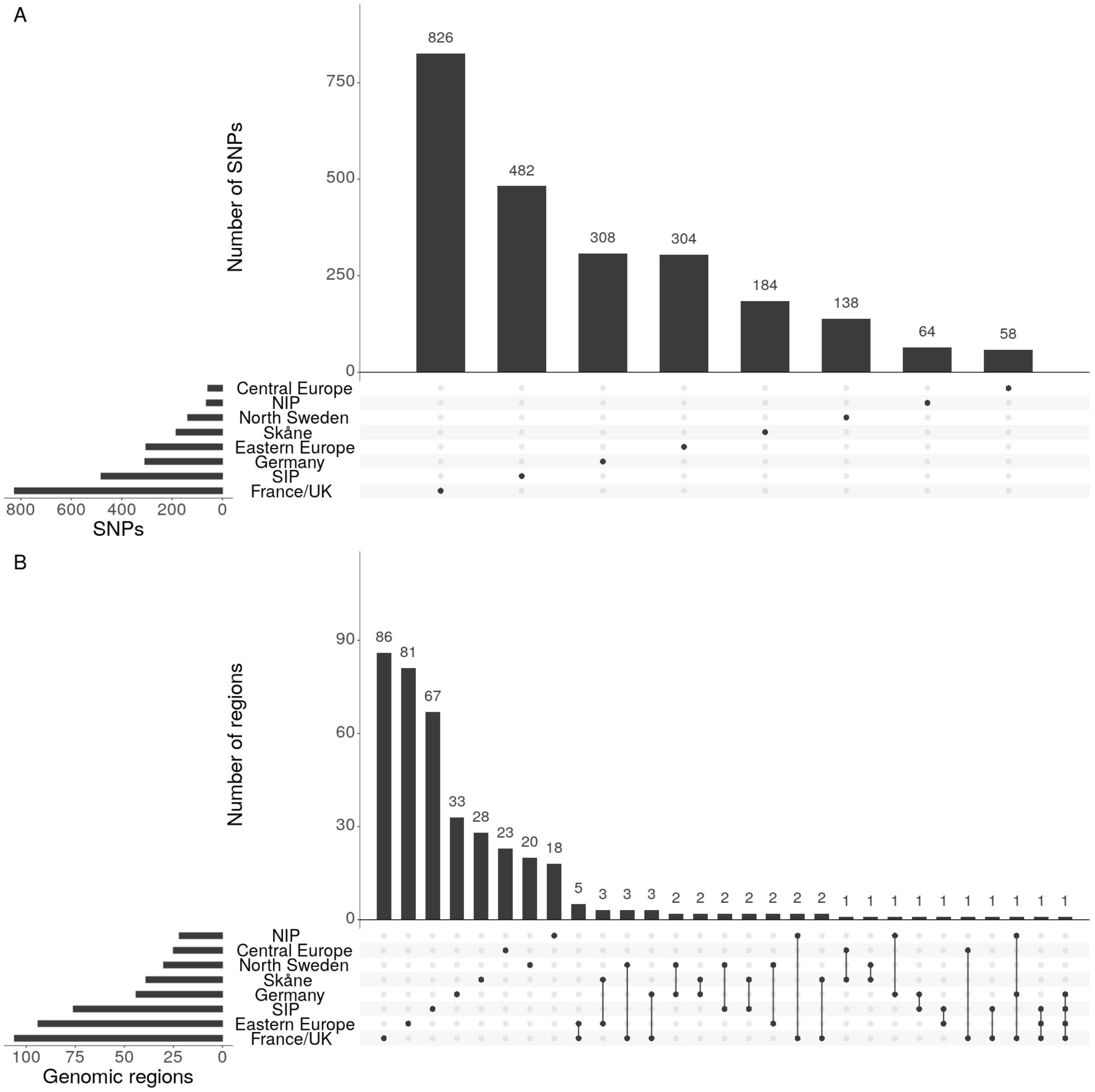

Supplementary Fig. 4: Sharing of sub-significant ($p < 10^{-4}$) associations for permuted phenotypes. (**A**) Histogram of the number of associated SNPs in each subpopulation and shared between pairs of subpopulations. (**B**) Histogram of the number of associated regions in each subpopulation and shared between pairs of subpopulations.

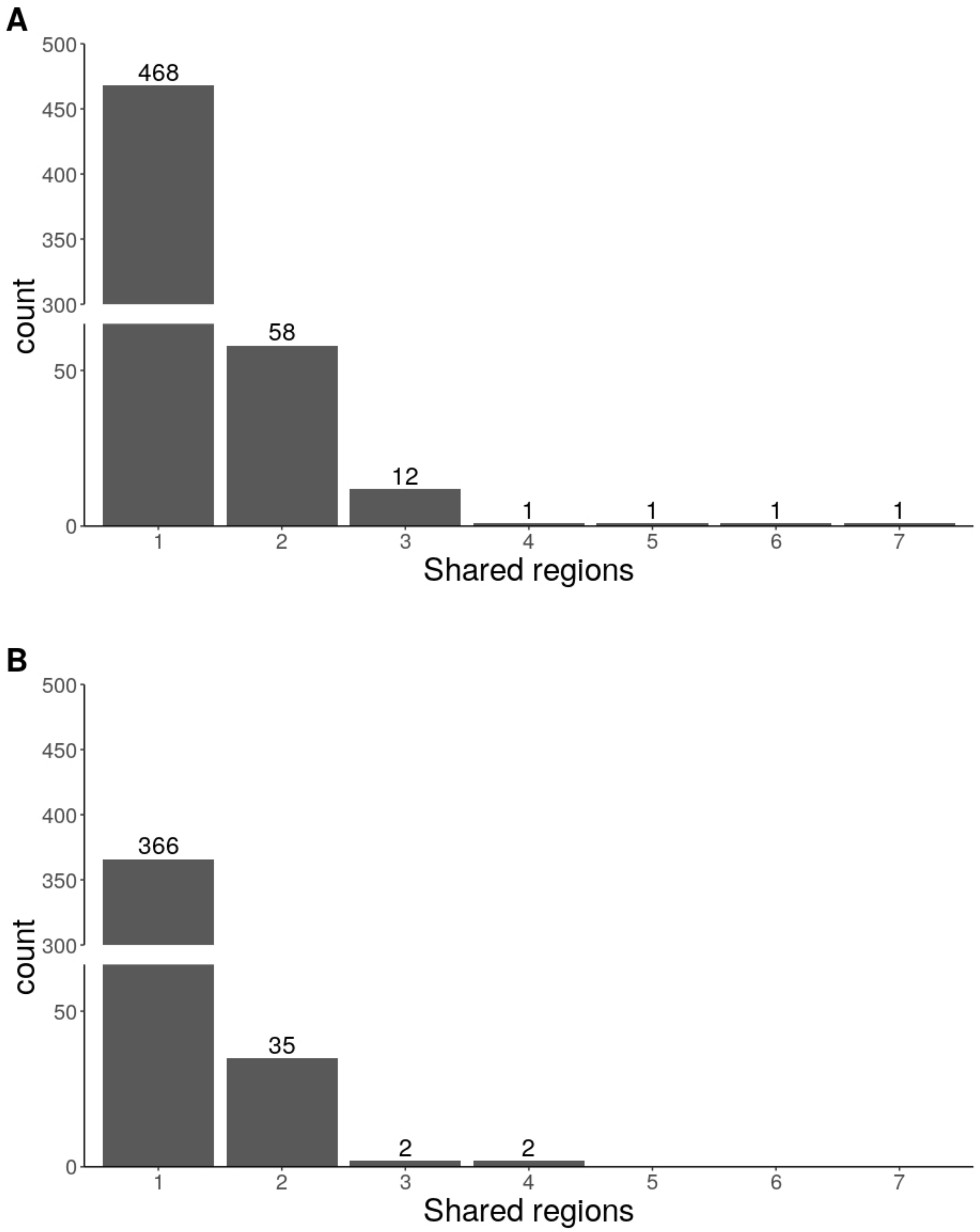

Supplementary Fig. 5: Bar plot showing the amount of regions that are associated across multiple subpopulations. (**A**) Analyses of FT10. (**B**) Analyses of permuted phenotypes.

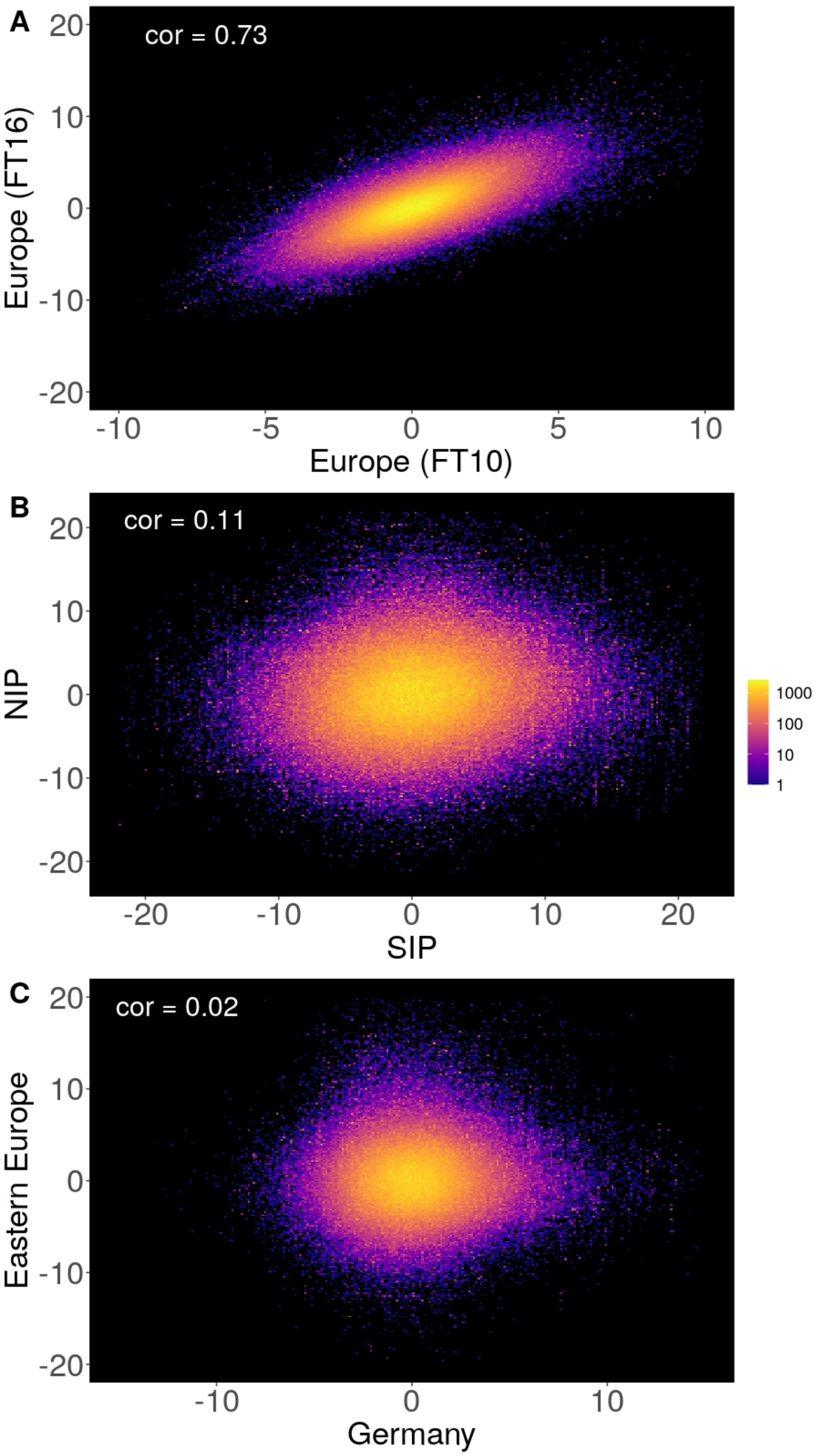

Supplementary Fig. 6: Density plots comparing effect sizes estimates using *MiXeR*. (**A**) The comparison between flowering time at 10°C and 16 °C in the complete European population. (**B**) Comparison of FT10 between NIP and SIP subpopulations. (**C**) Comparison of FT10 between the Eastern Europe and German subpopulations.

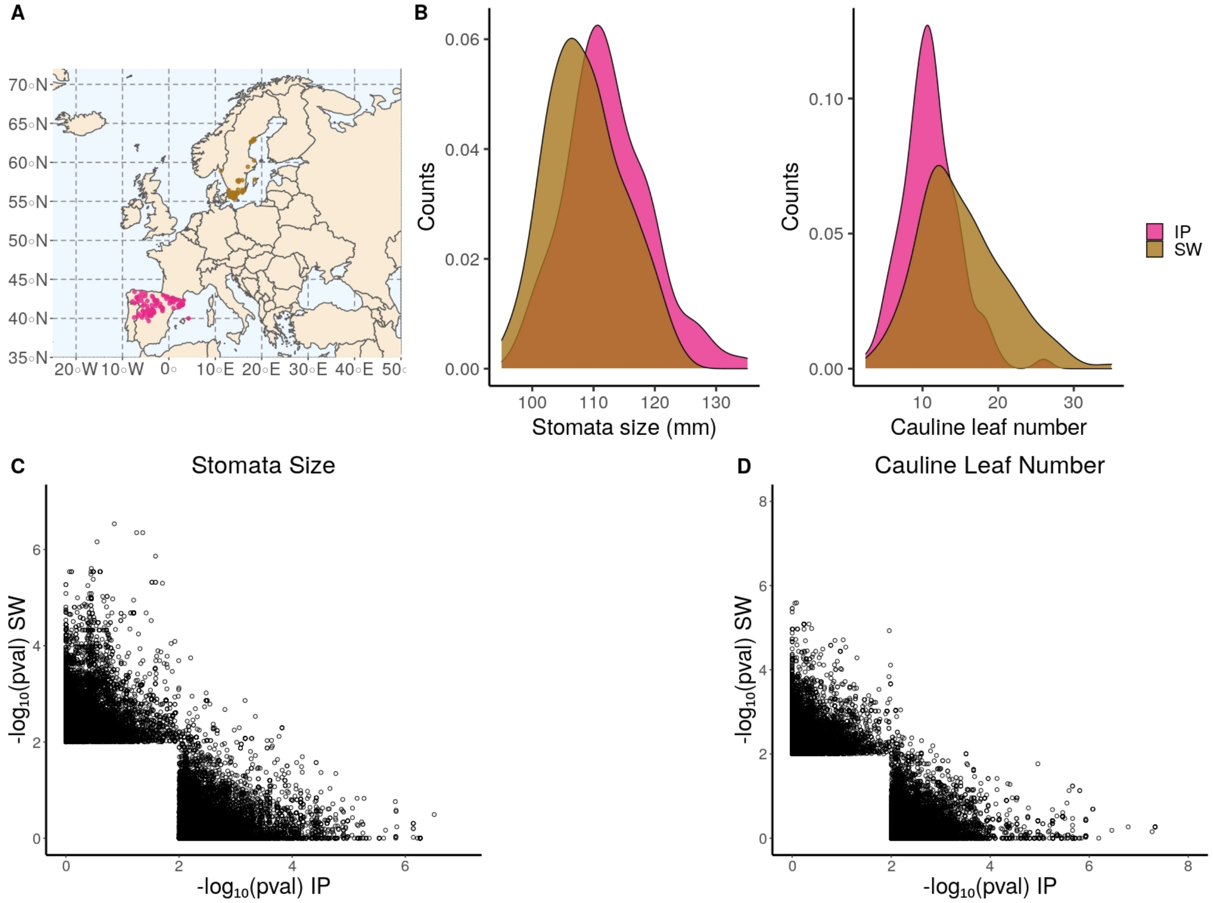

Supplementary Fig. 7: Analyses of stomata size and cauline leaf number.(**A**) Geographic distribution of the used 240 *A. thaliana* accessions.(**B**) Phenotypic distribution of stomata size and cauline leaf number in the Iberian (IP) and Swedish (SW) subpopulation.(**C-D**) Correlation plots of the -$\log_{10}$ p-values from the GWAS results obtain from the analyses within the different subpopulations for ST and CL. Marker with a p-value > 0.01 where removed for plotting from the respective subpopulation.

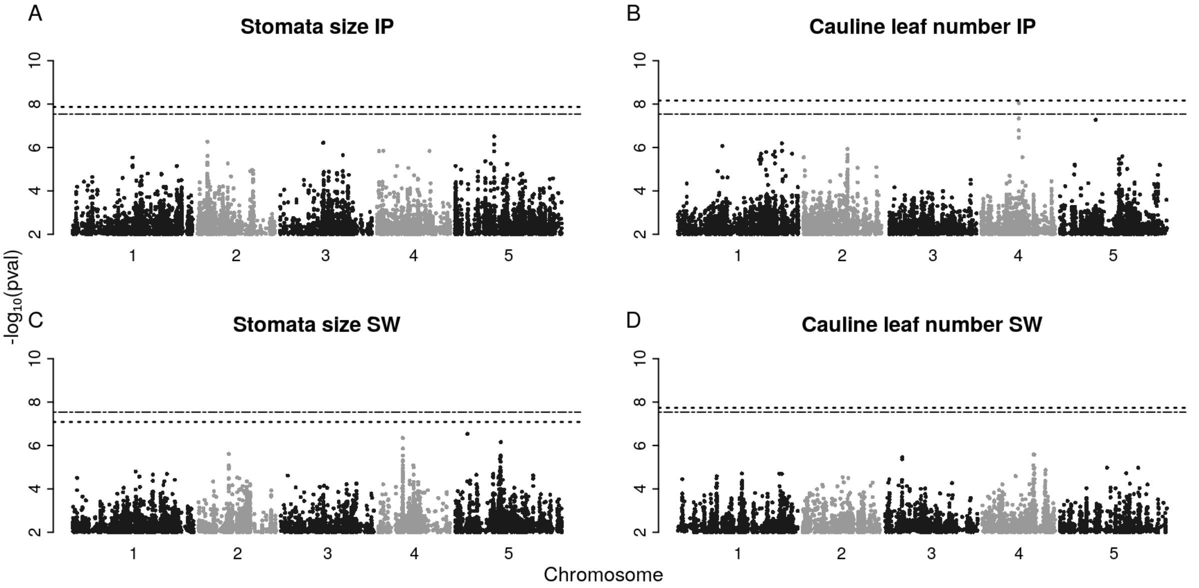

Supplementary Fig. 8: Manhattan plots of GWAS results from the analyses of ST and CL in the SW and IP subpopulations.

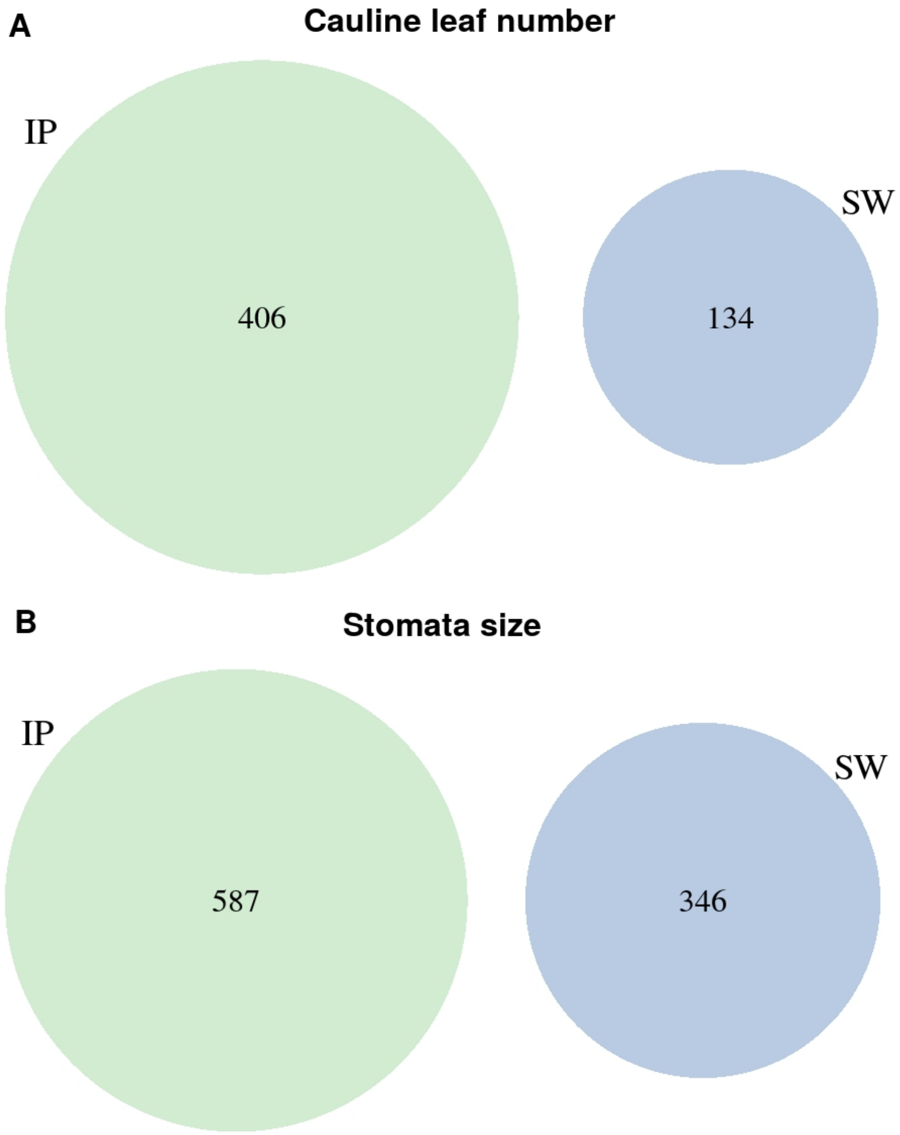

Supplementary Fig. 9: Venn diagram of associated markers ($p < 10^{-4}$) between the IP and SW subpopulations for ST (A) and CL (B).

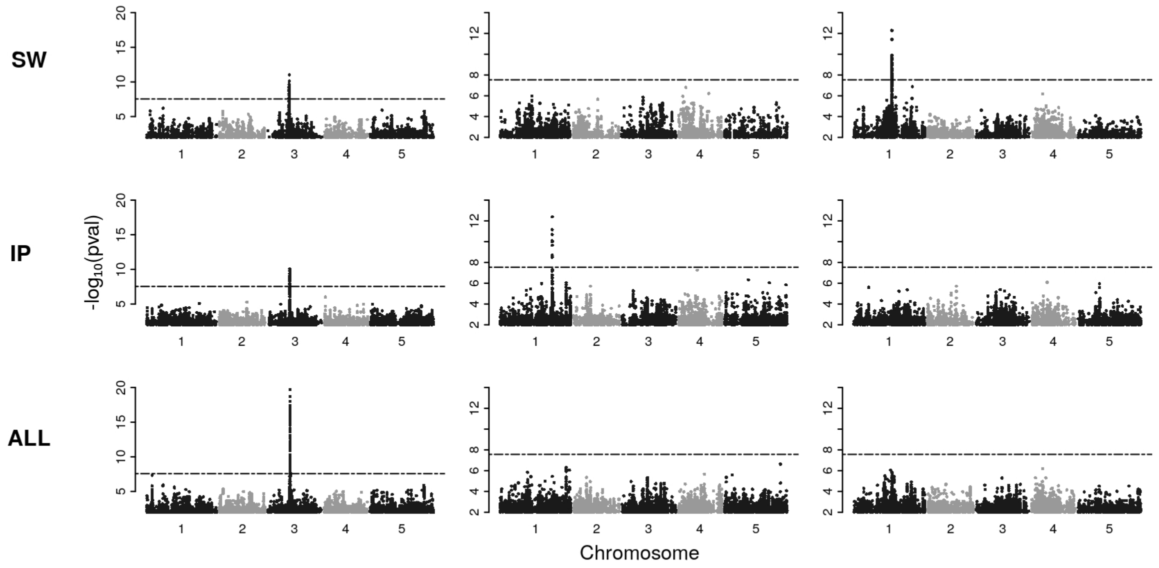

Supplementary Fig. 10: Manhattan plots of GWAS results from three different simulations. The causative markers were simulated to have an effect in all accessions (left panel), only in IP (middle panel) or only in SW (right panel). The respective population for GWAS are displayed in the different rows, where for the results in the top row, the SW subpopulation has been used, the IP subpopulation has been used to generate the results in the middle row and the bottom row displays the results in the merged population of 240 accessions. Dashed lines indicate the Bonferroni threshold used in the simulations.

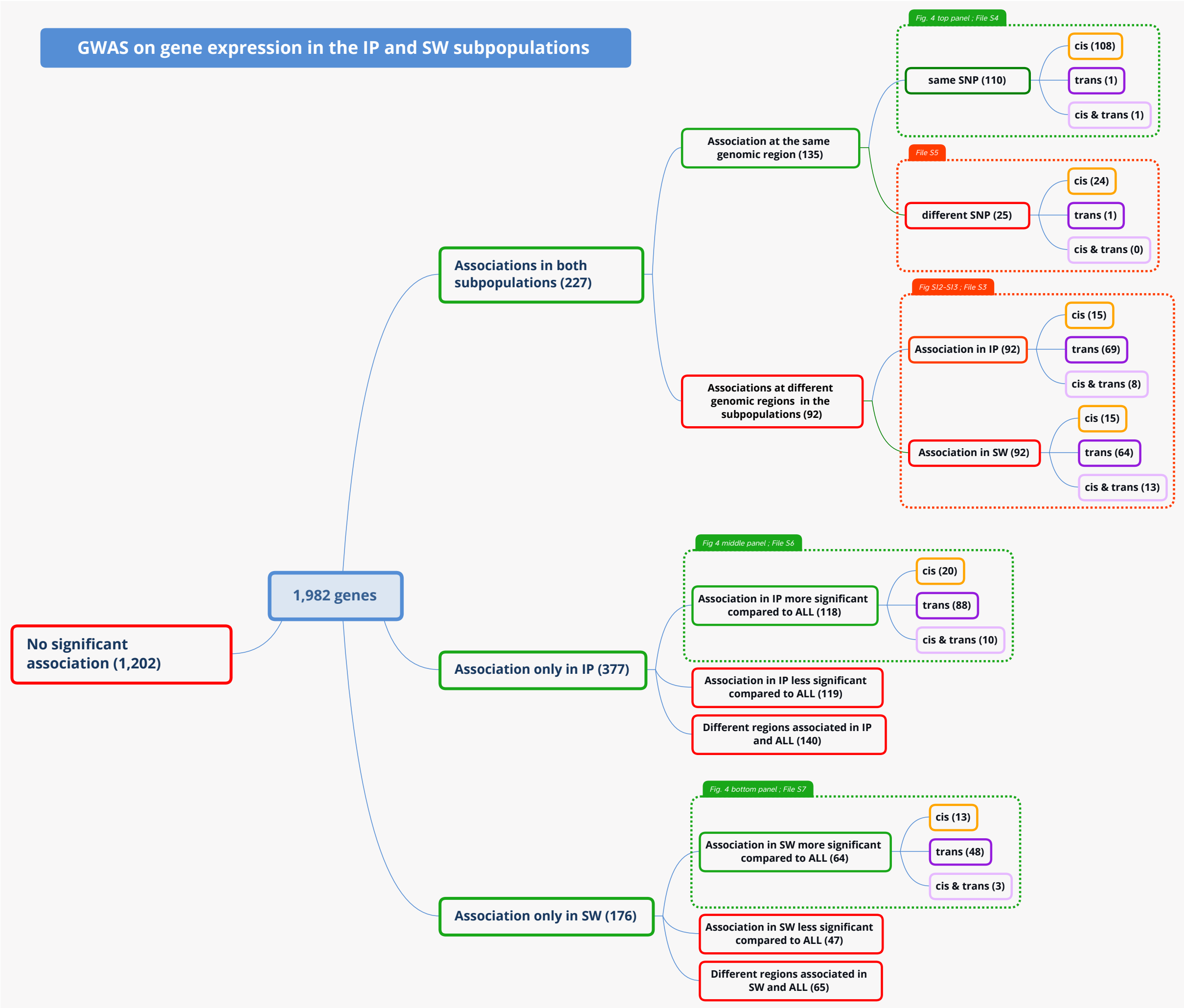

Supplementary Fig. 11: Graphical representation of the workflow and the different classes of associations found in GWAS on gene expression in the IP and SW subpopulations at a threshold of $p < 10^{-10}$.

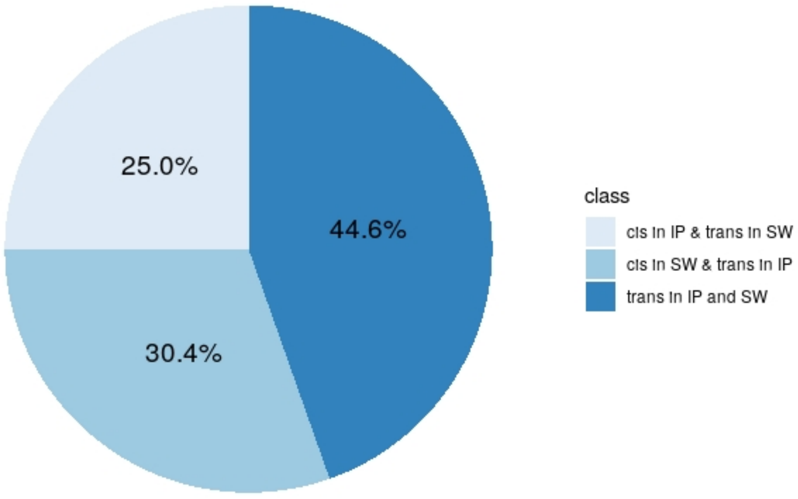

Supplementary Fig. 12: Summarized GWAS results for the analyses of RNA expression data in *A. thaliana* for genes that have associations at different regions in IP and SW. The pie chart displays the amount of genes having a *cis*-association in IP and a *trans*-association in SW, a *cis*-association in SW and a *trans*-association in IP or different *trans*-associations in both subpopulations.

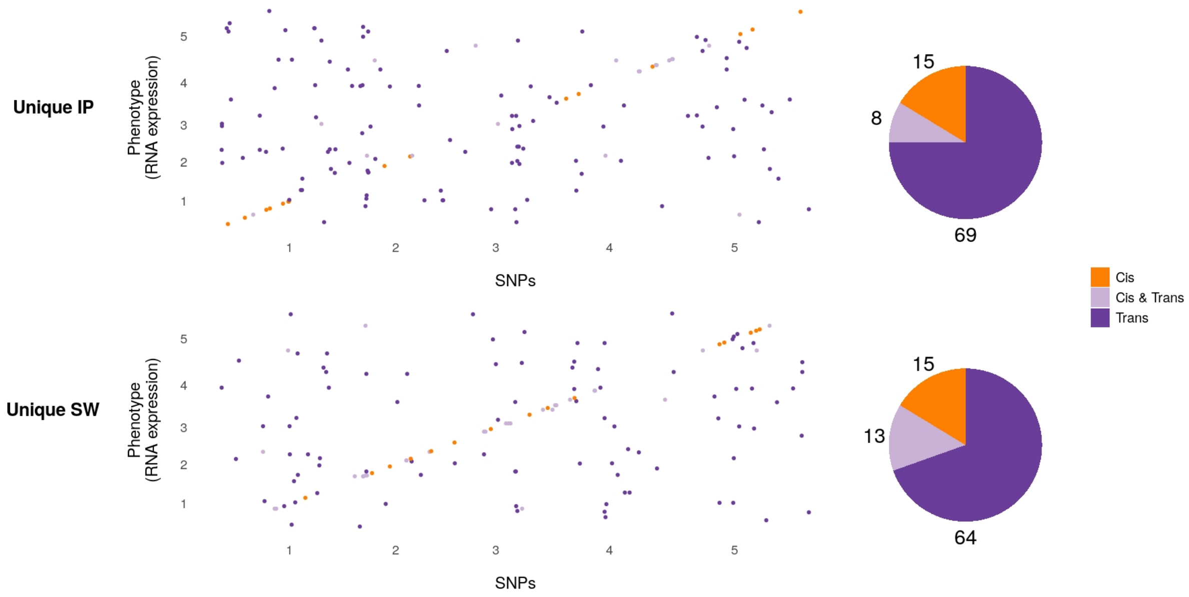

Supplementary Fig. 13: Summarized GWAS results for the analyses of RNA expression data in *A. thaliana* for genes that have associations at different regions in IP and SW. Scatter plots show the genomic location of the respective associated markers per gene. Top panel shows the amount of *cis* and *trans* associations in IP, and the bottom panel shows the location of the associations for the same genes in SW. *Cis*-regulatory variants are colored in orange, while variants in *trans* are shown in purple. Pie charts display the amount of genes per class that have *cis*, *cis* and *trans* or only *trans* associations.

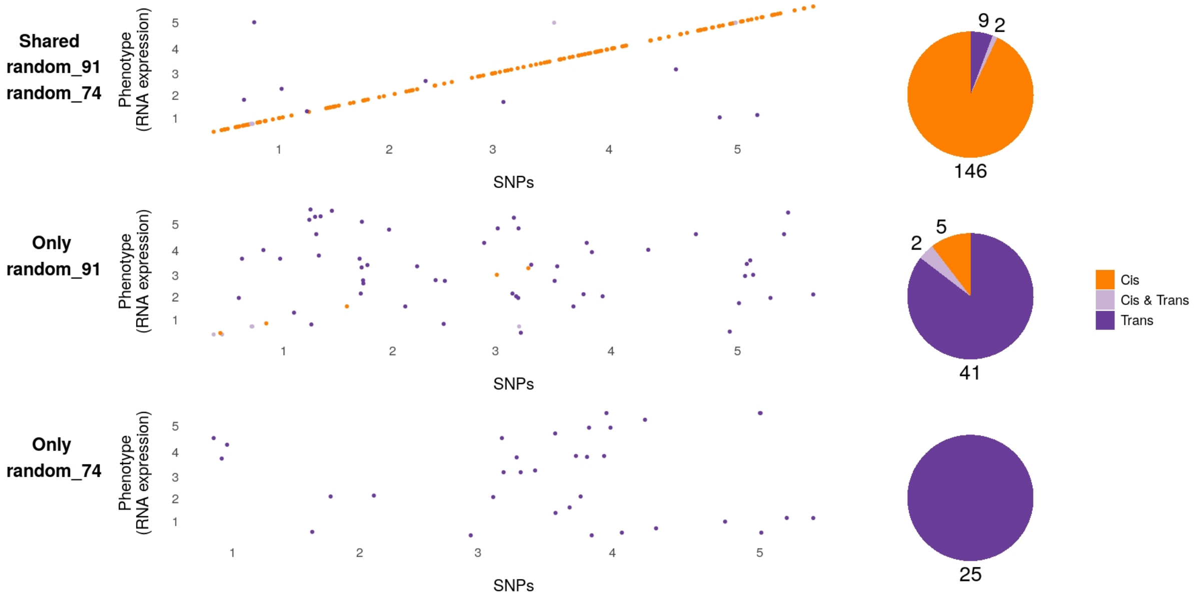

Supplementary Fig. 14: Summarized GWAS results for the analyses of RNA expression data in *A. thaliana* in random subpopulations. Genes are grouped in three categories: 1) Shared random_91/random_74, where the same association for a gene is recapitulated in the GWAS of both subpopulations. 2) Only random_91, where a significant association is only found using the random subpopulation containing 91 accessions. 3) Only random_74, where a significant association is only found using the random subpopulation containing 74 accessions. Scatter plots show the genomic location of the respective associated markers per gene for each class, where *cis*-regulatory variants are colored in orange, while variants in *trans* are shown in purple. Pie charts display the amount of genes per class that have *cis*, *cis* and *trans* or only *trans*-associations.

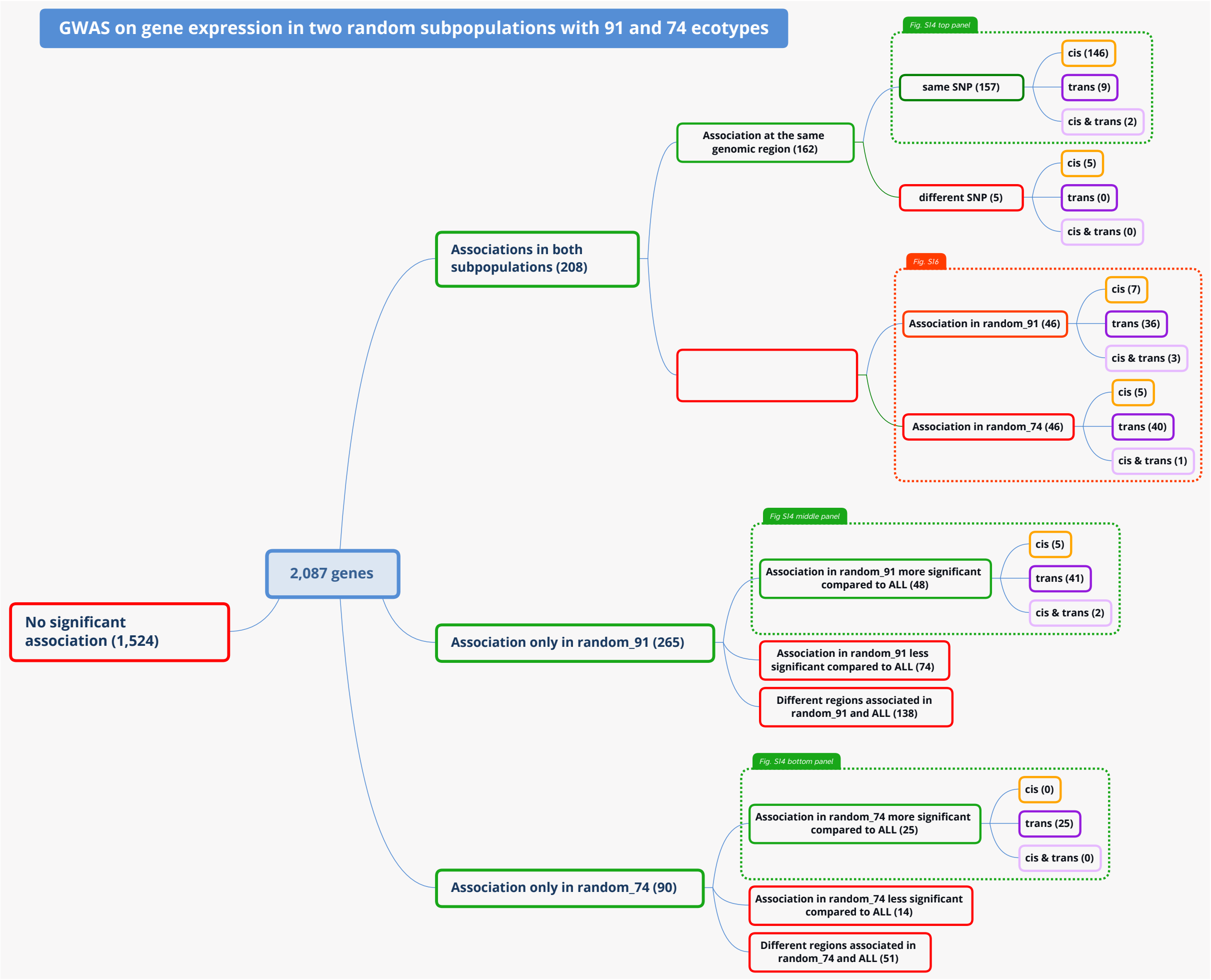

Supplementary Fig. 15: Graphical representation of of the different classes of associations found in GWAS on gene expression in two random subpopulations of 91 and 74 accessions at a threshold of $p < 10^{-10}$.

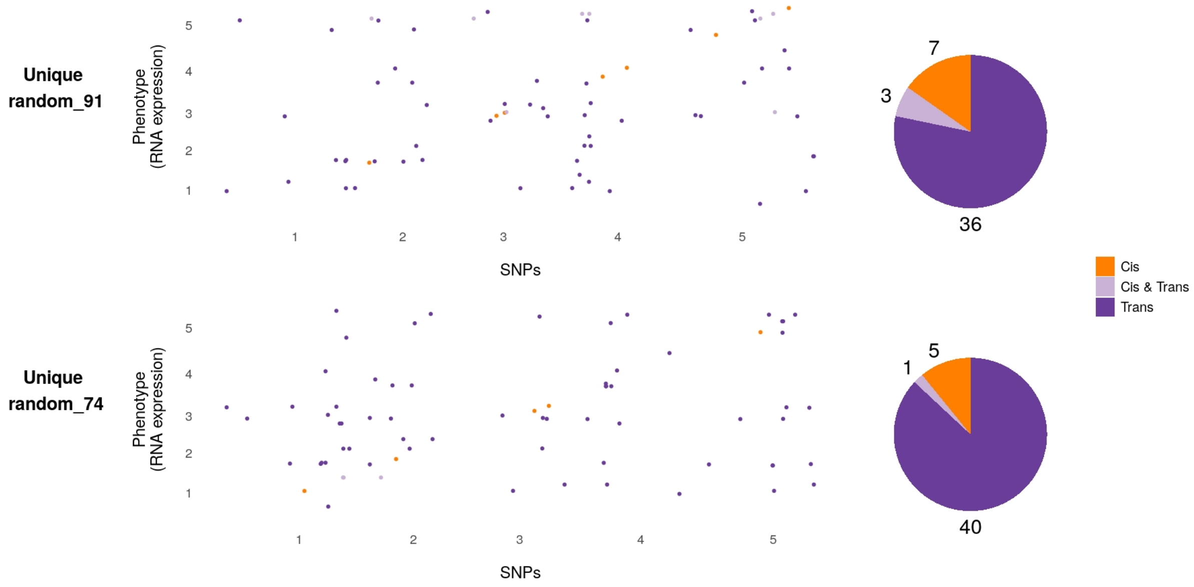

Supplementary Fig. 16: Summarized GWAS results for the analyses of RNA expression data in *A. thaliana* in random subpopulations for genes that have associations at different regions in both random subpopulations. Scatter plots show the genomic location of the respective associated markers per gene. Top panel shows the amount of *cis* and *trans*-association in random_91, and the bottom panel show the location of the associations for the same genes in random_74. *Cis*-regulatory variants are colored in orange, while variants in *trans* are shown in purple. Pie charts display the amount of genes per class that have *cis*, *cis* and *trans* or only *trans*-associations.

2 Supplementary Tables

Supplementary Table 1: Geographic location of the European subpopulations.

Subsets	No. accessions	min_lat	max_lat	min_lon	max_lon	$\widehat{h_2}$[a]	power
SIP	107	36.52	41.48	-8.54	4.25	0.99	1.00
NIP	108	41.50	47.45	-7.80	6.13	0.91	0.99
Germany	107	48.39	55.67	8.00	13.73	0.99	1.00
France/UK	107	47.50	57.97	-5.98	7.50	0.95	0.99
Central Europe	106	37.30	49.37	6.08	17.31	0.66	0.99
Skåne	119	55.38	56.10	13.10	14.78	0.91	0.99
Northern Sweden	118	56.10	68.80	6.19	18.52	0.75	0.99
Eastern Europe	116	37.07	61.36	38.28	38.28	0.91	0.99
Europe	888	36.52	68.80	-8.54	38.28	0.86	1.00

[a] $\widehat{h_2}$: pseudo-heritability estimate

Supplementary Table 2: Number of significant SNPs after Bonferroni and permutation-based threshold in the different subpopulations

Subsets	Bonferroni_Threshold	Sig_Bonferroni	Permutation_Threshold	Sig_Permutation
Europe	1.1e-08	29	1.7e-09	2
SIP	2.5e-08	33	2.6e-09	2
NIP	2.6e-08	4	1.4e-07	4
Germany	2.7e-08	14	1.6e-10	0
France and UK	2.8e-08	0	3.8e-12	0
Central Europe	2.6e-08	1	7.3e-08	12
Skåne	2.7e-08	0	4.6e-08	0
Northern Sweden	2.7e-08	26	1.2e-08	2
Eastern Europe	2.6e-08	1	1.3e-10	0

Supplementary Table 3: P-value and minor allele frequency of the SNPs that are significant in the European set (after Permutation-based threshold) in the different subpopulations

subpopulation	1:24339560[a]	10 kb window[b]		5:18590501[a]	10 kb window[b]	
		SNP	Pval (MAF)		SNP	Pval (MAF)
Europa	2.39e-10 (0.44)			1.71e-09 (0.20)		
SIP	1.68e-02 (0.45)	1:24339614	9.88e-04 (0.30)	2.93e-02 (0.28)	5:18605212	3.54e-03 (0.47)
NIP	1.25e-02 (0.35)	1:24338003	1.40e-04 (0.30)	1.83e-08 (0.14)	5:18590501	1.83e-08 (0.14)
Germany	3.08e-02 (0.28)	1:24346295	2.82e-04 (0.07)	2.58e-02 (0.06)	5:18565932	1.49e-03 (0.17)
France/UK	6.99e-04 (0.41)	1:24342759	4.41e-05 (0.40)	1.43e-01 (0.05)	5:18606201	1.00e-03 (0.4)
Central Europe	7.40e-02 (0.46)	1:24329854	6.70e-03 (0.05)	8.15e-02 (0.05)	5:18590327	5.20e-03 (0.34)
Skåne	5.1e-02 (0.24)	1:24320374	9.34e-03 (0.27)	1.51e-01 (0.33)	5:18585844	2.38e-03 (0.07)
Northern Sweden	3.11e-01 (0.20)	1:24348634	1.89e-02 (0.15)	1.03e-01 (0.48)	5:18590741	1.19e-04 (0.14)
Eastern Europe	2.61e-01 (0.44)	1:24325815	3.47e-03 (0.17)	7.43e-08 (0.08)	5:18590501	7.43e-08 (0.08)

[a] "chromosome:position"
[b] the most significant marker in a 10 kb window around the lead SNP is reported for each subpopulation

Supplementary Table 4: Shared SNPs between subpopulations at significance level of $p < 10^{-4}$

Associated marker[a]	1:29186215	1:29199833	3:20379636	4:6781375	5:18589998	5:18590247	5:18590501	5:18590591
Candidate gene[b]			*SMZ*[c]		*DOG1*[d]	*DOG1*[d]	*DOG1*[d]	*DOG1*[d]
SIP	3.75e-05 (0.06)	3.75e-05 (0.06)	6.22e-01 (0.04)	1.15e-02 (0.02)	1.38e-01 (0.04)	2.92e-02 (0.03)	2.92e-02 (0.03)	2.92-02 (0.03)
NIP	4.80e-01 (0.12)	4.80e-01 (0.12)	2.51e-01 (0.06)	9.45e-01 (0.06)	1.83e-08 (0.14)	1.83e-08 (0.14)	1.83e-08 (0.14)	1.83e-08 (0.14)
Germany	1.55e-01 (0.10)	1.04e-01 (0.10)	9.06e-05 (0.07)	3.88e-01 (0.07)	8.28e-03 (0.05)	2.73e-01 (0.05)	2.58e-02 (0.05)	6.30e-01 (0.03)
France/UK	7.34e-01 (0.29)	7.34e-01 (0.29)	4.52e-01 (0.20)	7.75e-05 (0.08)	1.74e-03 (0.019)	1.86e-02 (0.03)	1.43e-01 (0.05)	5.53e-03 (0.03)
Central Europe	5.90e-01 (0.03)	5.9e-01 (0.03)		2.70e-02 (0.13)	2.60e-01 (0.05)	4.60e-01 (0.05)	8.15e-02 (0.05)	
Skåne	8.55e-01 (0.25)	8.55e-01 (0.25)	4.35e-02 (0.10)	4.44e-01 (0.45)	8.00e-01 (0.23)	9.86e-02 (0.32)	1.51e-01 (0.33)	1.80e-01 (0.37)
Northern Sweden	4.61e-01 (0.5)	4.78e-01 (0.5)	4.49e-01 (0.43)	7.04e-01 (0.36)	5.12e-01 (0.34)	4.59e-01 (0.48)	1.03e-01 (0.48)	5.75e-10 (0.36)
Eastern Europe	2.85e-05 (0.05)	2.85e-05 (0.05)	9.29e-05 (0.05)	9.62e-05 (0.06)	7.43-08 (0.08)	1.05e-06 (0.09)	7.43e-08 (0.08)	7.43e-08 (0.08)

[a] represented as "chromosome:position"
[b] known flowering time gene within a 10 kb window around the associated marker using a list of 306 flowering time genes from Bouché et al. 2016
[c] *SMZ (SCHLAFMÜTZE)*, Mathieu et al. 2009
[d] *DOG1 (DELAY OF GERMINATION)*, Huo et al. 2016

Supplementary Table 5: Overlap of candidate genes with shared genomic regions

Region[a]	1:(26151612–26570642)	4:(192421–572878)	5:(22786643 23605491)
Candidate gene[b]	*CDF5*[c]	*FRI*[d]*LIF2*[e]*MED12*[f]	*COL5*[g]*MSI1*[h]*VIN3*[i] *ZTL*[j]
SIP	0	0	1
NIP	1	1	1
Germany	1	1	1
France/UK	1	1	1
Central Europe	1	0	1
Skåne	0	1	0
North Sweden	1	1	1
East Europe	1	0	1

[a] represented as "chromosome:(start–stop)
[b] known flowering time gene within the detected region using a list of 306 flowering time genes from Bouché et al. 2016
[c] *CDF5 (CYCLING DOF FACTOR 5)*, Fornara et al. 2009
[d] *FRI (FRIGIDA)*, Stinchcombe et al. 2004
[e] *LIF2 (LHP1-INTERACTING FACTOR 2)*, Latrasse et al. 2011
[f] *MED12 (MEDIATOR 12)*, Imura et al. 2012
[g] *COL5 (CONSTANS-LIKE 5)*, Hassidim et al. 2009
[h] *MSI (MULTICOPY SUPRESSOR OF IRA1)*, Bouveret et al. 2006
[i] *VIN3 (VERNALIZATION INSENSITIVE 3)*, Sung and Amasino 2004
[j] *ZTL (ZEITLUPE)*, Kim et al. 2007

Supplementary Table 6: Estimation of shared causal variants and effect size correlation using *MIXeR*

subpopulation 1	subpopulation 2	shared variants[a]	rho_shared[b]	rho_all[c]
SIP	NIP	122	0.81	0.11
SIP	Germany	29	0.42	0.05
SIP	France/UK	1	NA[d]	0.04
SIP	North Sweden	26	-0.26	0.02
SIP	Eastern Europe	52	-0.20	0.02
NIP	Germany	116	-0.85	0.02
NIP	France/UK	1	NA	0.06
NIP	North Sweden	29	-0.13	0.02
NIP	Eastern Europe	76	0.31	0.03
Germany	France/UK	14	.89	0.08
Germany	North Sweden	31	0.90	0.02
Germany	Eastern Europe	32	-0.59	0.02
France/UK	North Sweden	27	-0.01	0.05
France/UK	Eastern Europe	1	NA	0.07
North Sweden	Eastern Europe	26	0.58	0.07
FT10	FT16	154	0.95	0.73

[a] estimated number of shared causal variants
[b] estimated effect size correlation between shared causal variants
[c] estimated effect size correlation between all variants from marginal GWAS results
[d] as only one shared variant has been estimated, the correlation of the effect size of this variants is not possible
The models for combination containing either the Central European or the Skaane subpopulations didn't converge, therefore these subpopulations have been excluded from the analyses.

Supplementary Table 7: Geographic limits, pseudo-heritability and power estimation of the IP and SW subpopulation used for the analyses of stomata size (ST) and cauline leaf number (CL).

Trait	Source	No. accessions	min_lat	max_lat	min_lon	max_lon	$\widehat{h_2}$	Power
ST	ALL	240	39.66	63.02	-7.80	18.52	0.56	1.00
	IP	109	39.66	43.40	-7.80	4.25	0.27	0.81
	SW	131	55.38	63.02	11.2	18.52	0.54	0.99
CL	ALL	240	39.66	63.02	-7.80	18.52	0.78	1.00
	IP	109	39.66	43.40	-7.80	4.25	0.9	1.00
	SW	131	55.38	63.02	11.2	18.52	0.85	1.00

Supplementary Table 8: Summarized GWAS results from simulated data. True positives (TP), false positives (FP) and false discovery rate (FDR) are reported for 1,000 simulations per scenario with GWAS performed either in all 240 accessions (ALL) or only in the IP or SW subpopulation.

Variance explained		TP			FP			FDR		
		ALL	IP	SW	ALL	IP	SW	All	IP	SW
20%	ALL	964	264	390	225	73	146	0.19	0.22	0.27
	IP	270	274	0	135	73	4	0.33	0.21	1
	SW	65	0	420	187	0	156	0.74	NA	0.27

Supplementary Table 9: Overview of the sensitivity of the different simulation scenarios.

Variance explained		Sensitivity		
		ALL	IP	SW
	ALL	0.265	0.130	0.250
15%	IP	0.050	0.000	0.230
	SW	0.020	0.140	0.000
	ALL	0.170	0.000	0.20
10%	IP	0.010	0.000	0.020
	SW	0.000	0.000	0.000
	ALL	0.000	0.000	0.000
5%	IP	0.000	0.000	0.000
	SW	0.000	0.000	0.000

Supplementary Table 10: Overview of RNA expression data. This table shows the filters applied to the whole data. GWAS was performed on 2,483 genes.

Filter	Sample size
Total RNA expression data	24,175
Nuclear genes	23,021
$\widehat{h_2} > 0.5$	4,873
$\widehat{h_2} > 0.5$ & power > 0.9	2,483
$\widehat{h_2} > 0.5$ & power > 0.9 and not inflated	1,982

3 Supplementary Files

Suppl. File 1 : Shared regions in the analyses of flowering time and associated candidate genes.
Suppl. File 2 : Summary of the number of associated SNPs and regions for GWAS on 2,237 gene expression data.
Suppl. File 3 : List of 92 genes, which show different associations in the GWAS on the RNAseq data in the two subpopulation.
Suppl. File 4 : List of 110 genes, which show the same association in the RNAseq analysis in IP and SW.
Suppl. File 5 : List of 25 genes, which show an association in the RNAseq analysis in IP and SW subpopulation at the same region, but with different associated markers.
Suppl. File 6 : List of 118 genes, which show an association in the GWAS of the RNAseq data only in the IP subpopulation.
Suppl. File 7 : List of 64 genes, which show an association in the GWAS of the RNAseq data only in the SW subpopulation.
All files are accessible via https://github.com/arthurkorte/genetic_heterogeneity

References

Bouché F, Lobet G, Tocquin P, Périlleux C. 2016. Flor-id: an interactive database of flowering-time gene networks in arabidopsis thaliana. *Nucleic Acids Research*, 44(D1):D1167–D1171.

Bouveret R, Schönrock N, Gruissem W, Hennig L. 2006. Regulation of flowering time by arabidopsis msi1. *Development*, 133(9):1693–1702.

Fornara F, Panigrahi K. C, Gissot L, Sauerbrunn N, Rühl M, Jarillo J. A, Coupland G. 2009. Arabidopsis dof transcription factors act redundantly to reduce constans expression and are essential for a photoperiodic flowering response. *Developmental Cell*, 17(1):75–86.

Hassidim M, Harir Y, Yakir E, Kron I, Green R. M. 2009. Over-expression of constans-like 5 can induce flowering in short-day grown arabidopsis. *Planta*, 230(3):481–491.

Huo H, Wei S, Bradford K. J. 2016. Delay of germination1 (dog1) regulates both seed dormancy and flowering time through microrna pathways. *Proceedings of the National Academy of Sciences*, 113(15): E2199–E2206.

Imura Y, Kobayashi Y, Yamamoto S, Furutani M, Tasaka M, Abe M, Araki T. 2012. Cryptic precocious/med12 is a novel flowering regulator with multiple target steps in arabidopsis. *Plant and Cell Physiology*, 53(2):287–303.

Kim W.-Y, Fujiwara S, Suh S.-S, Kim J, Kim Y, Han L, David K, Putterill J, Nam H. G, Somers D. E. 2007. Zeitlupe is a circadian photoreceptor stabilized by gigantea in blue light. *Nature*, 449(7160): 356–360.

Latrasse D, Germann S, Houba-Hérin N, Dubois E, Bui-Prodhomme D, Hourcade D, Juul-Jensen T, Le Roux C, Majira A, Simoncello N et al. 2011. Control of flowering and cell fate by lif2, an rna binding partner of the polycomb complex component lhp1. *PloS one*, 6(1):e16592.

Mathieu J, Yant L. J, Mürdter F, Küttner F, Schmid M. 2009. Repression of flowering by the miR172 target SMZ. *PLoS Biology*, 7(7):e1000148.

Stinchcombe J. R, Weinig C, Ungerer M, Olsen K. M, Mays C, Halldorsdottir S. S, Purugganan M. D, Schmitt J. 2004. A latitudinal cline in flowering time in arabidopsis thaliana modulated by the flowering time gene frigida. *Proceedings of the National Academy of Sciences*, 101(13):4712–4717.

Sung S, Amasino R. M. 2004. Vernalization in arabidopsis thaliana is mediated by the phd finger protein vin3. *Nature*, 427(6970):159–164.
