# Supplementary material for: Global Genetic Heterogeneity in Adaptive Traits
Source: Mol Biol Evol. 2021 Jul 8;38(11):4822–31. doi: 10.1093/molbev/msab208 (PMC8557469; doi:10.1093/molbev/msab208)
Supplement: msab208_Supplementary_Data [file msab208_supplementary_data.zip › Supp_11.pdf]

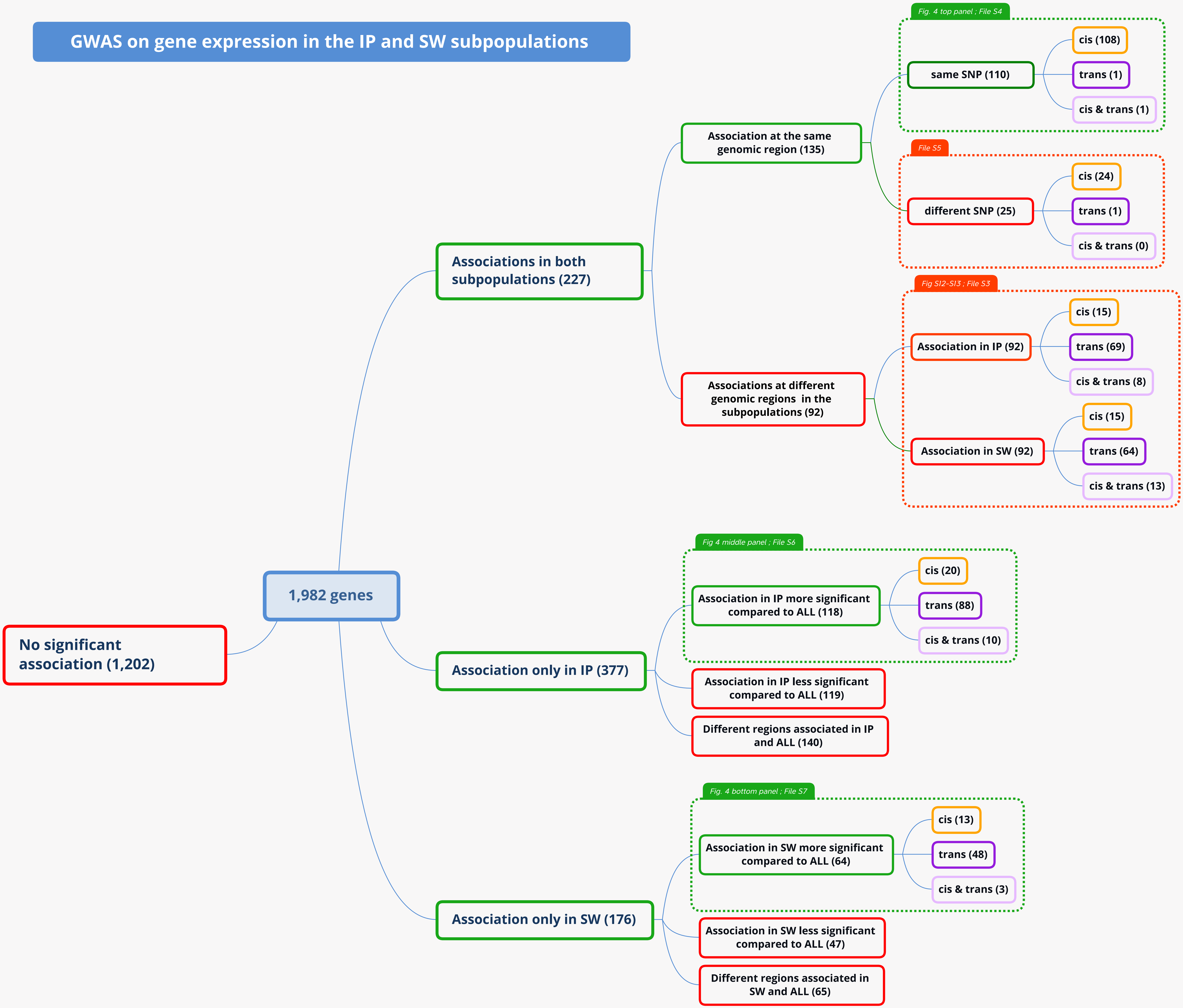
GWAS on gene expression in the IP and SW subpopulations
1,982 genes
No significant association (1,202)
Associations in both subpopulations (227)
Association at the same genomic region (135)
Fig. 4 top panel ; File S4
same SNP (110)
cis (108)
trans (1)
cis & trans (1)
File S5
different SNP (25)
cis (24)
trans (1)
cis & trans (0)
Associations at different genomic regions in the subpopulations (92)
Fig S12-S13 ; File S3
Association in IP (92)
cis (15)
trans (69)
cis & trans (8)
Association in SW (92)
cis (15)
trans (64)
cis & trans (13)
Association only in IP (377)
Fig 4 middle panel ; File S6
Association in IP more significant compared to ALL (118)
cis (20)
trans (88)
cis & trans (10)
Association in IP less significant compared to ALL (119)
Different regions associated in IP and ALL (140)
Association only in SW (176)
Fig. 4 bottom panel ; File S7
Association in SW more significant compared to ALL (64)
cis (13)
trans (48)
cis & trans (3)
Association in SW less significant compared to ALL (47)
Different regions associated in SW and ALL (65)
