# Supplementary figures and images for: Global Genetic Heterogeneity in Adaptive Traits
Source: Mol Biol Evol. 2021 Jul 8;38(11):4822–31. doi: 10.1093/molbev/msab208 (PMC8557469; doi:10.1093/molbev/msab208)

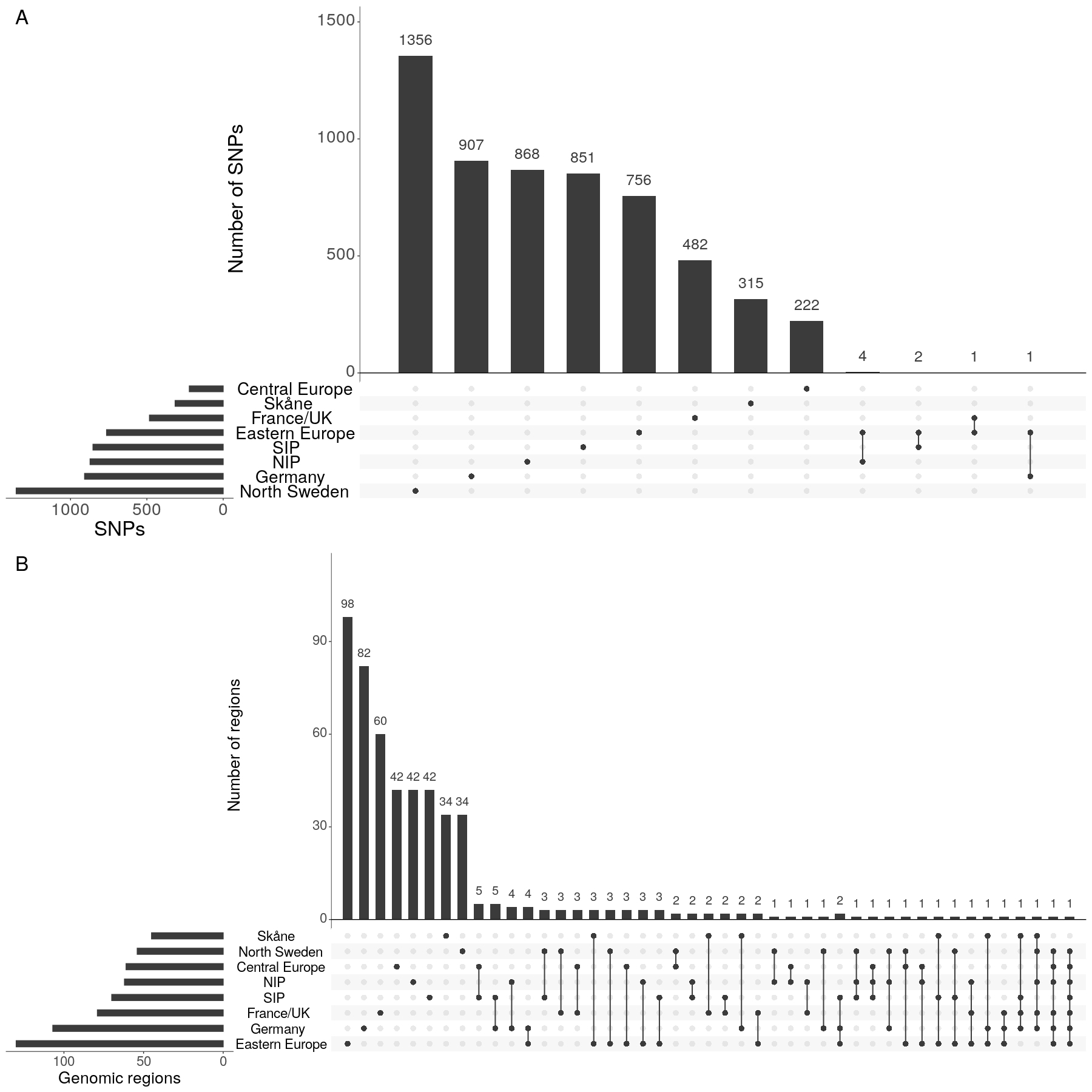

Supplement: msab208_Supplementary_Data [file msab208_supplementary_data.zip › Figura_2.jpeg]

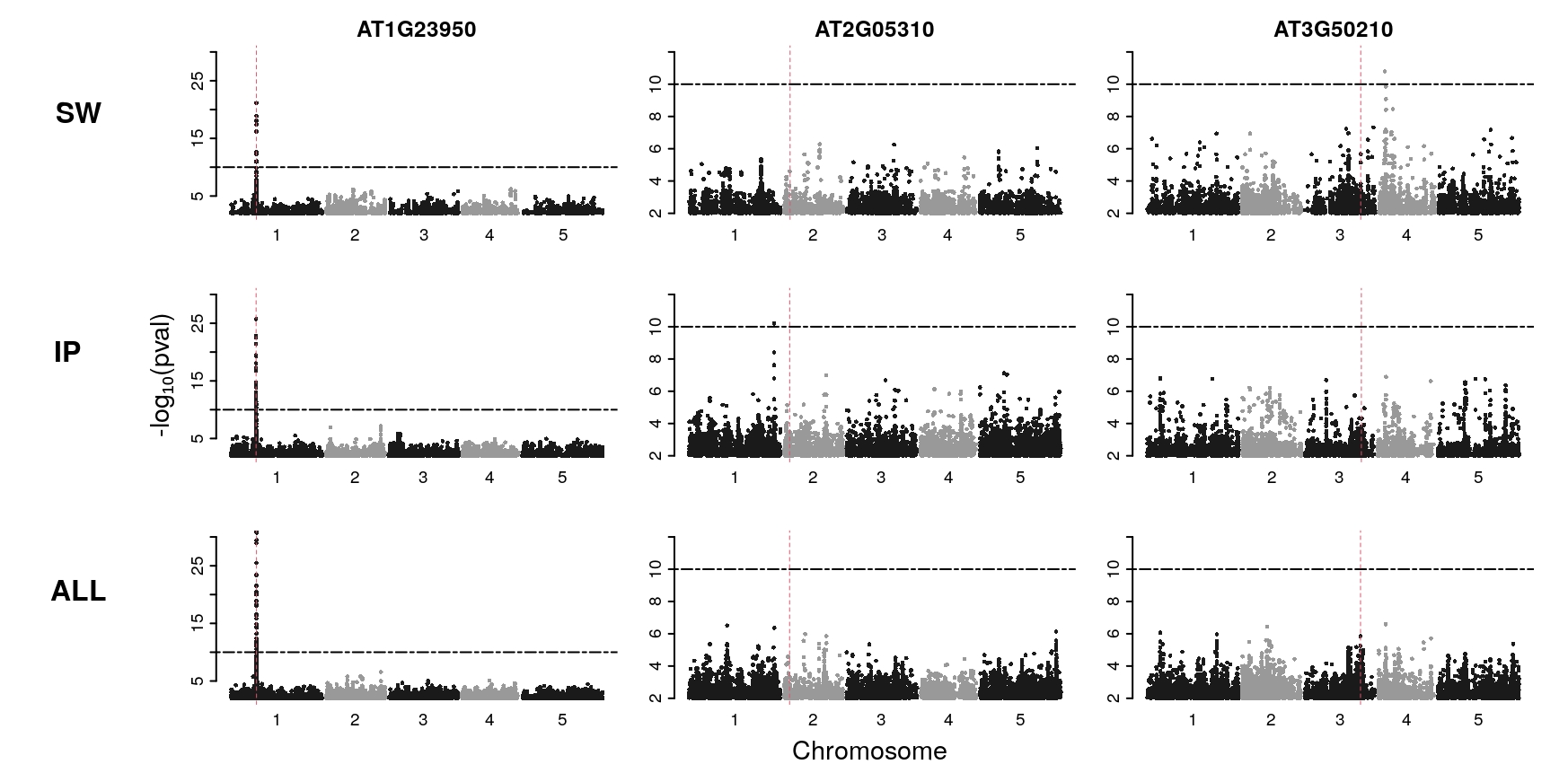

Supplement: msab208_Supplementary_Data [file msab208_supplementary_data.zip › Figura_3.jpeg]

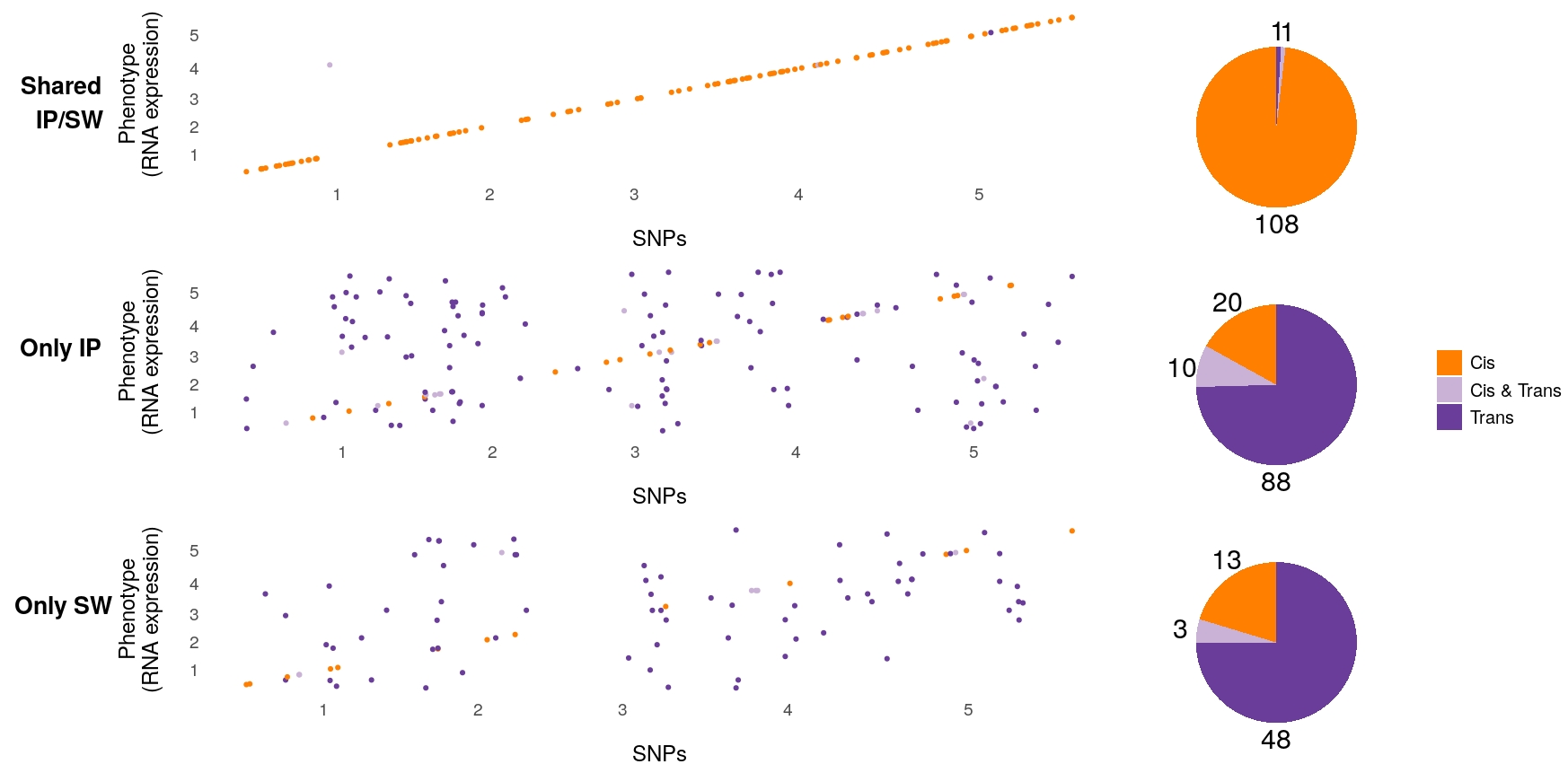

Supplement: msab208_Supplementary_Data [file msab208_supplementary_data.zip › Figura_4.jpeg]

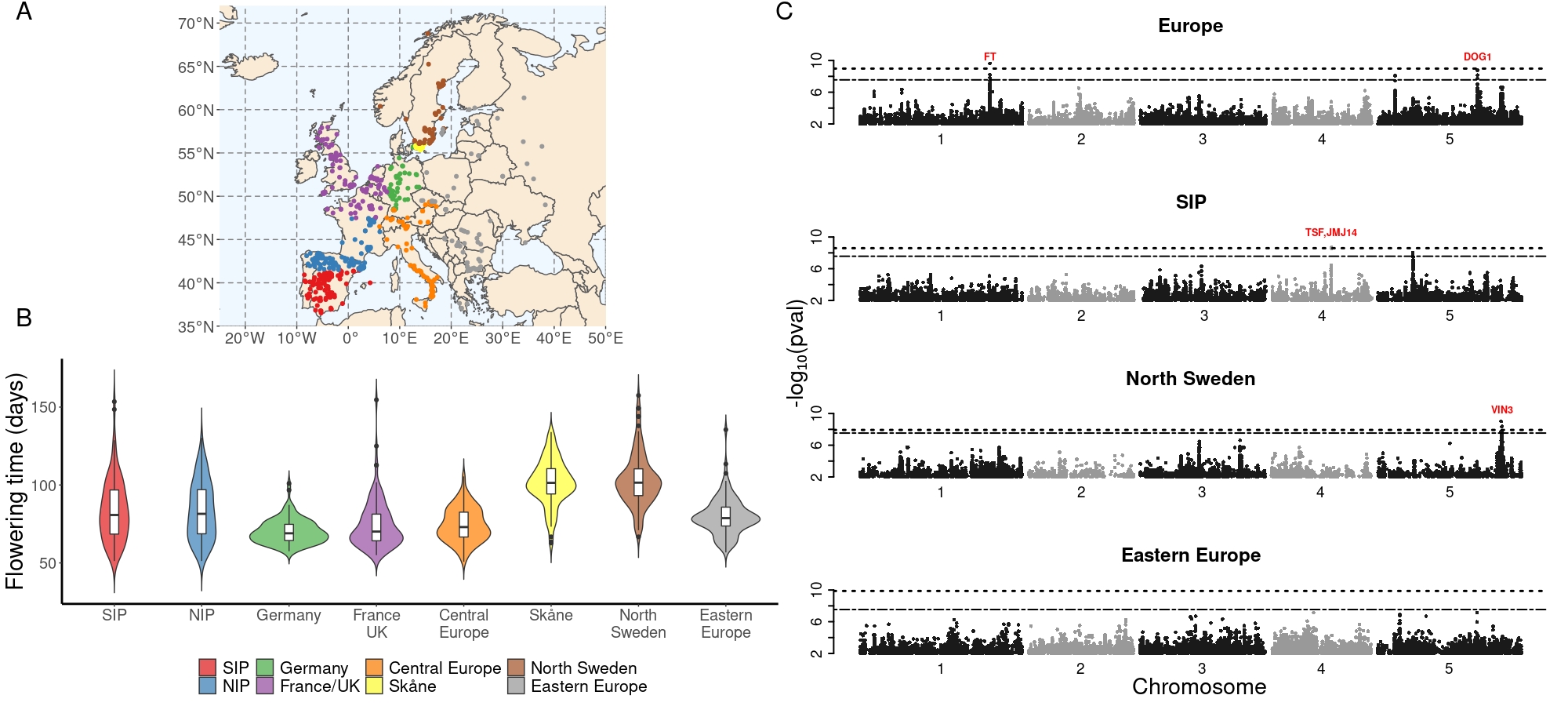

Supplement: msab208_Supplementary_Data [file msab208_supplementary_data.zip › Figura1.jpeg]

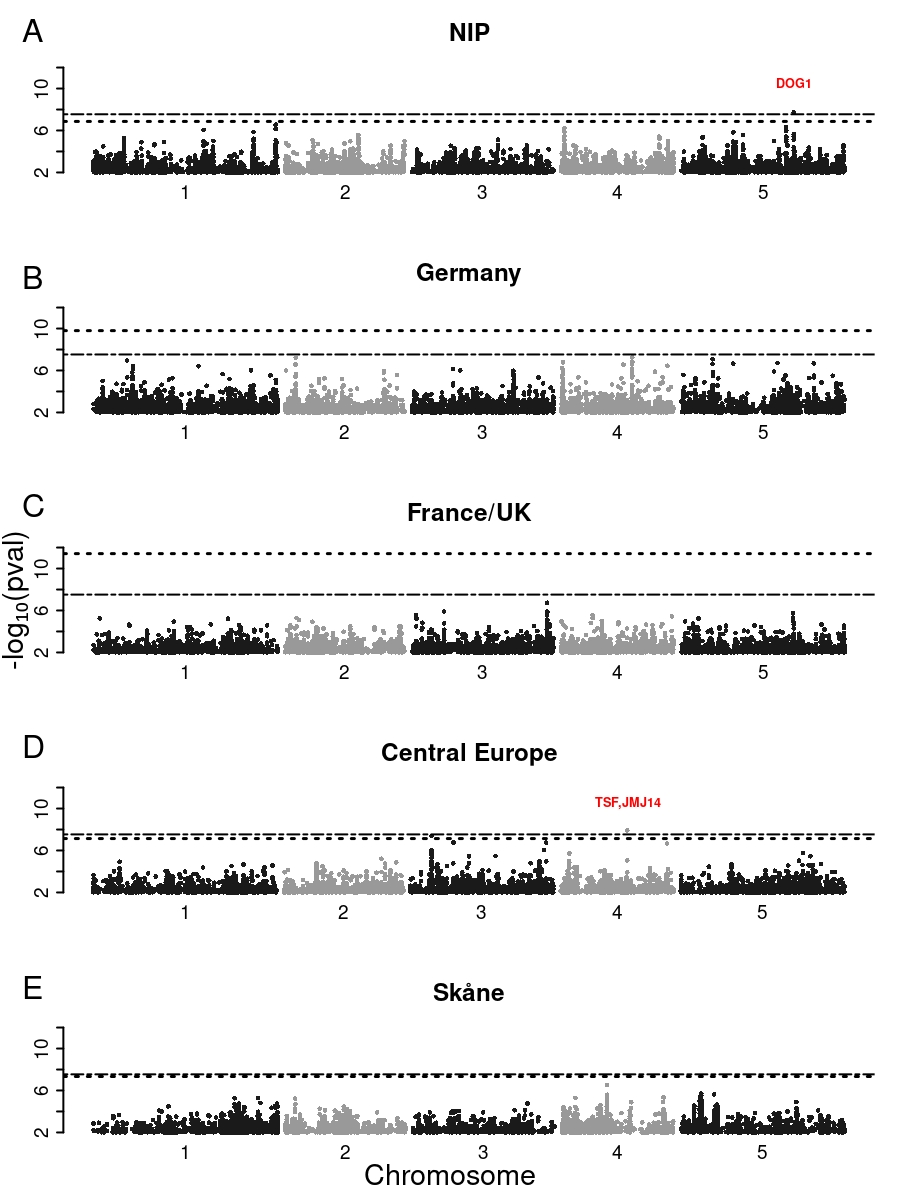

Supplement: msab208_Supplementary_Data [file msab208_supplementary_data.zip › Supp_1.jpeg]

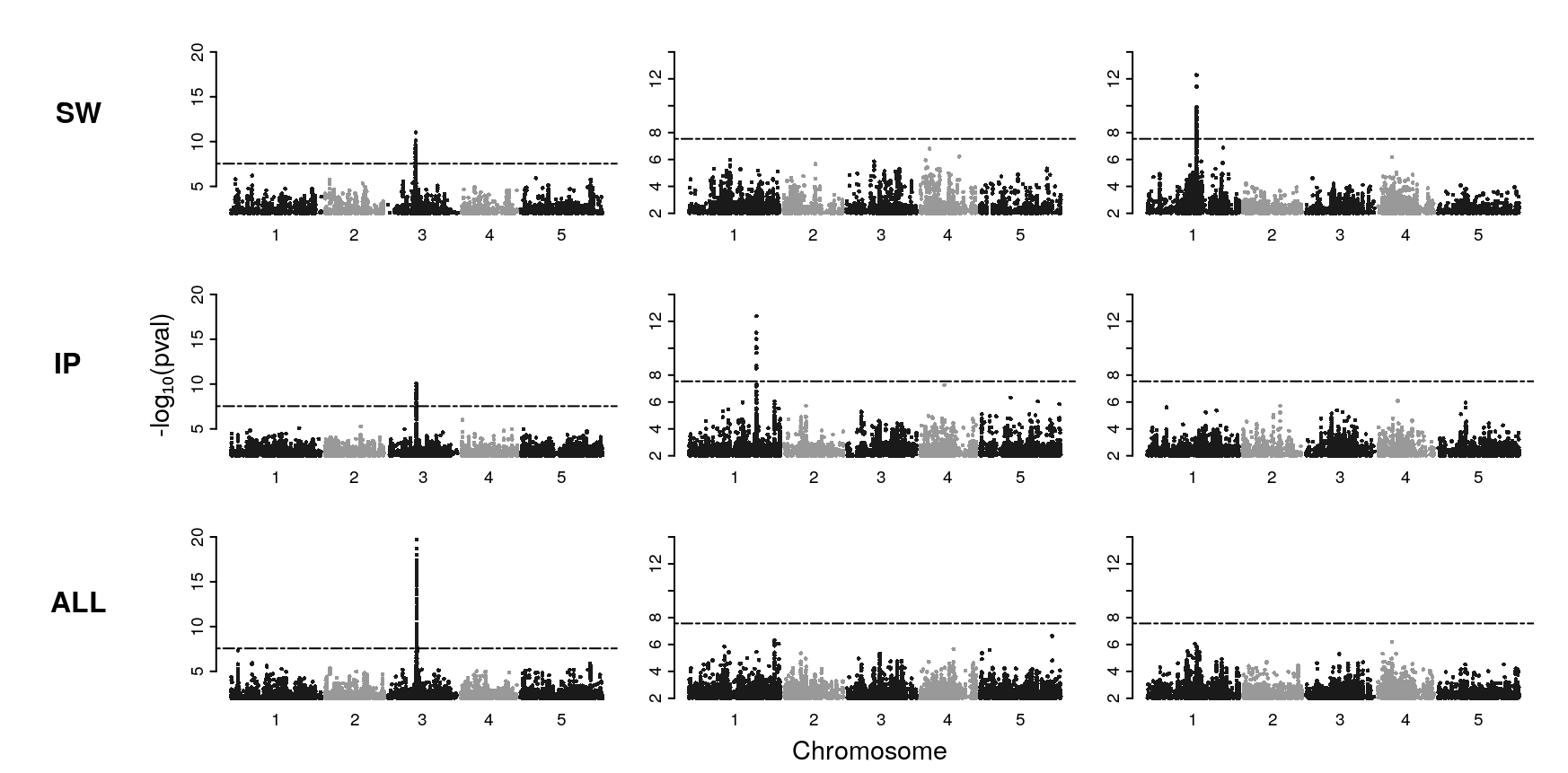

Supplement: msab208_Supplementary_Data [file msab208_supplementary_data.zip › Supp_10.jpeg]

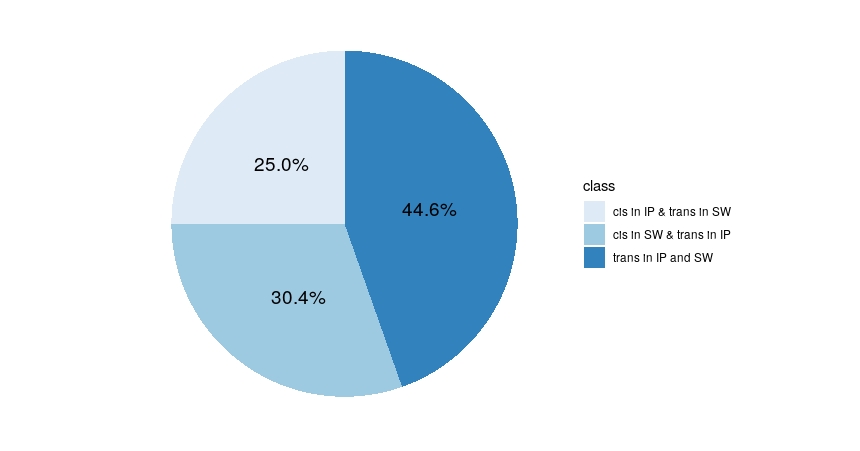

Supplement: msab208_Supplementary_Data [file msab208_supplementary_data.zip › Supp_12.jpeg]

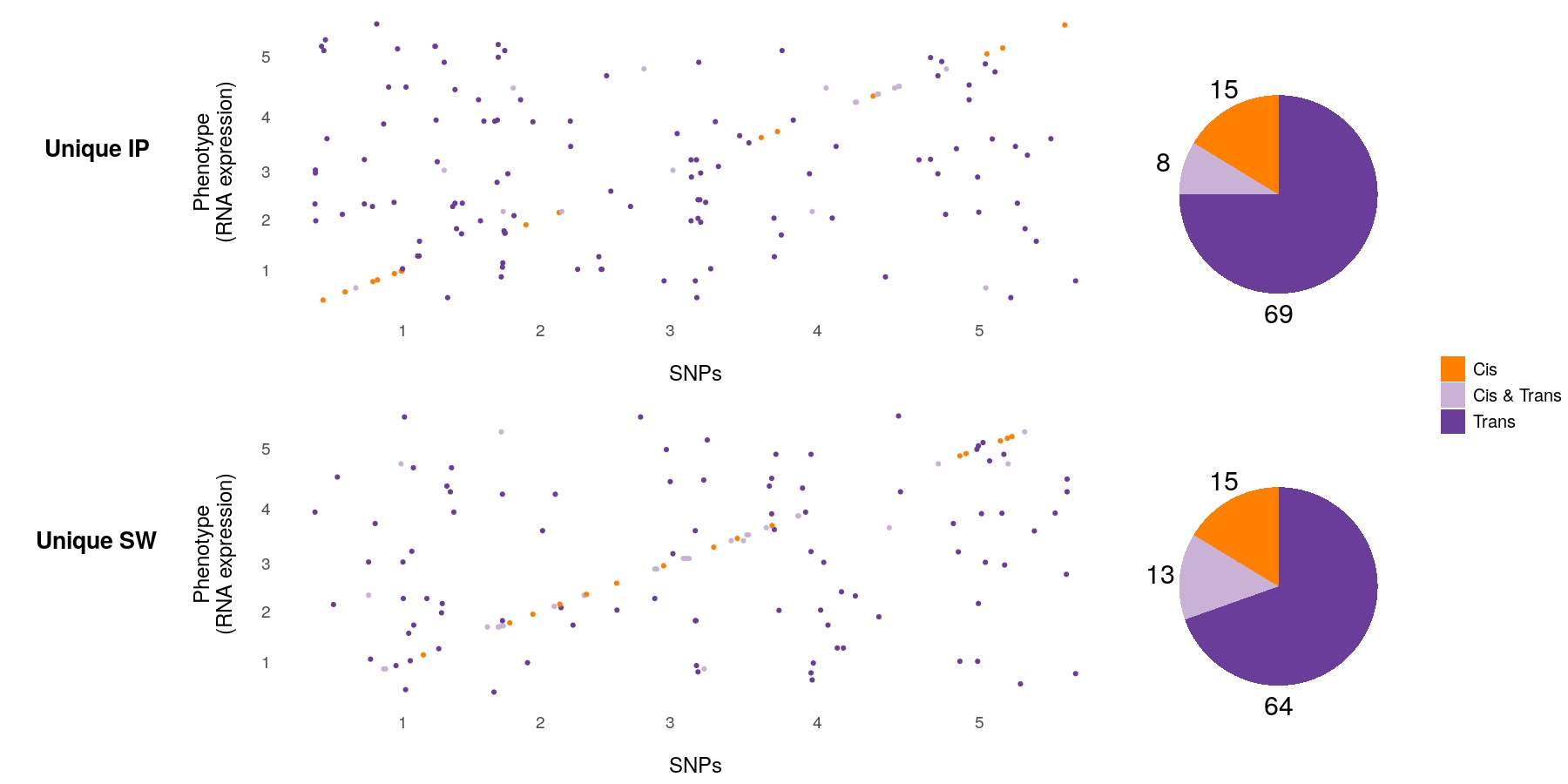

Supplement: msab208_Supplementary_Data [file msab208_supplementary_data.zip › Supp_13.jpeg]

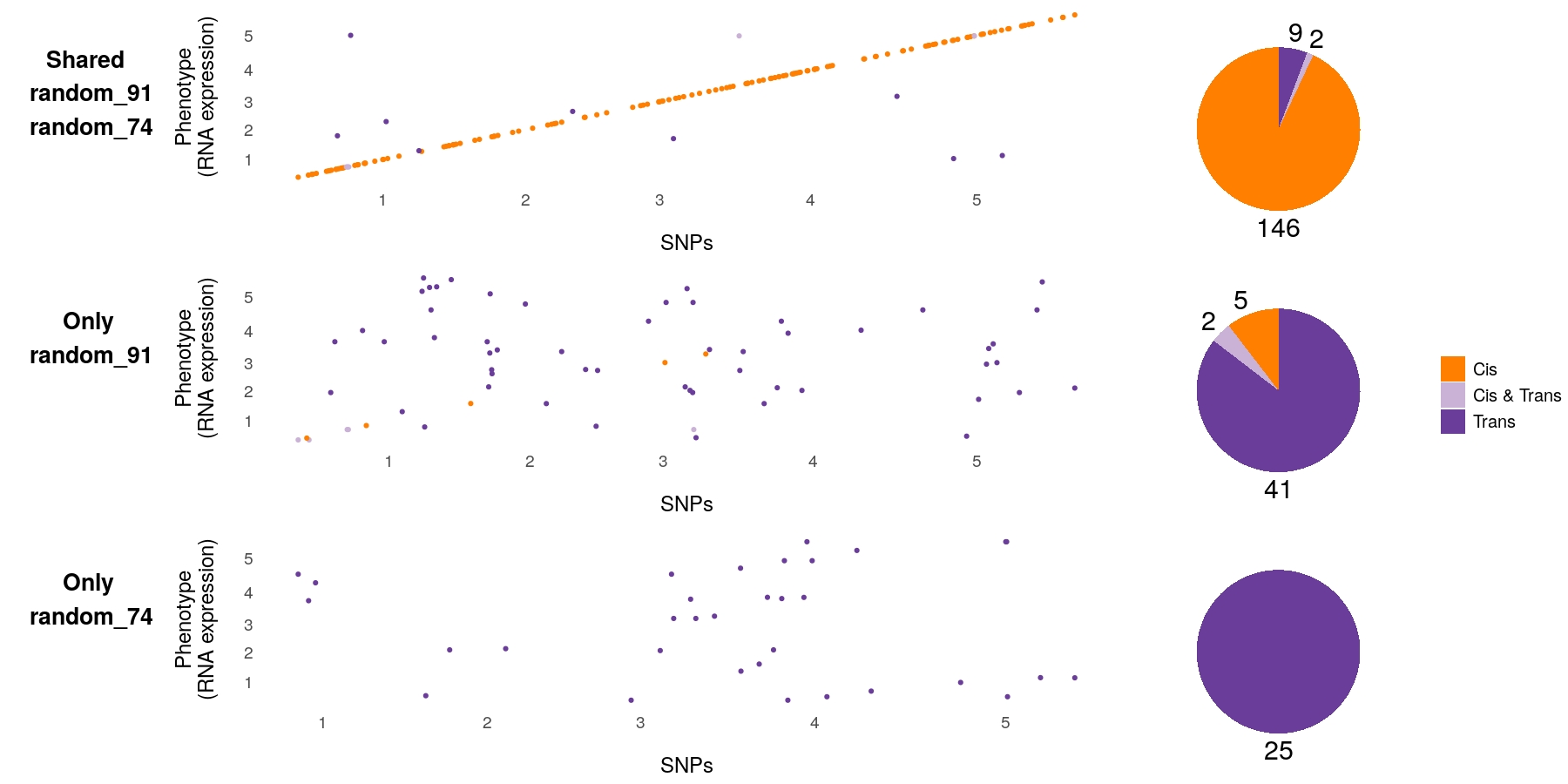

Supplement: msab208_Supplementary_Data [file msab208_supplementary_data.zip › Supp_14.jpeg]

GWAS on gene expression in two random subpopulations with 91 and 74 ecotypes

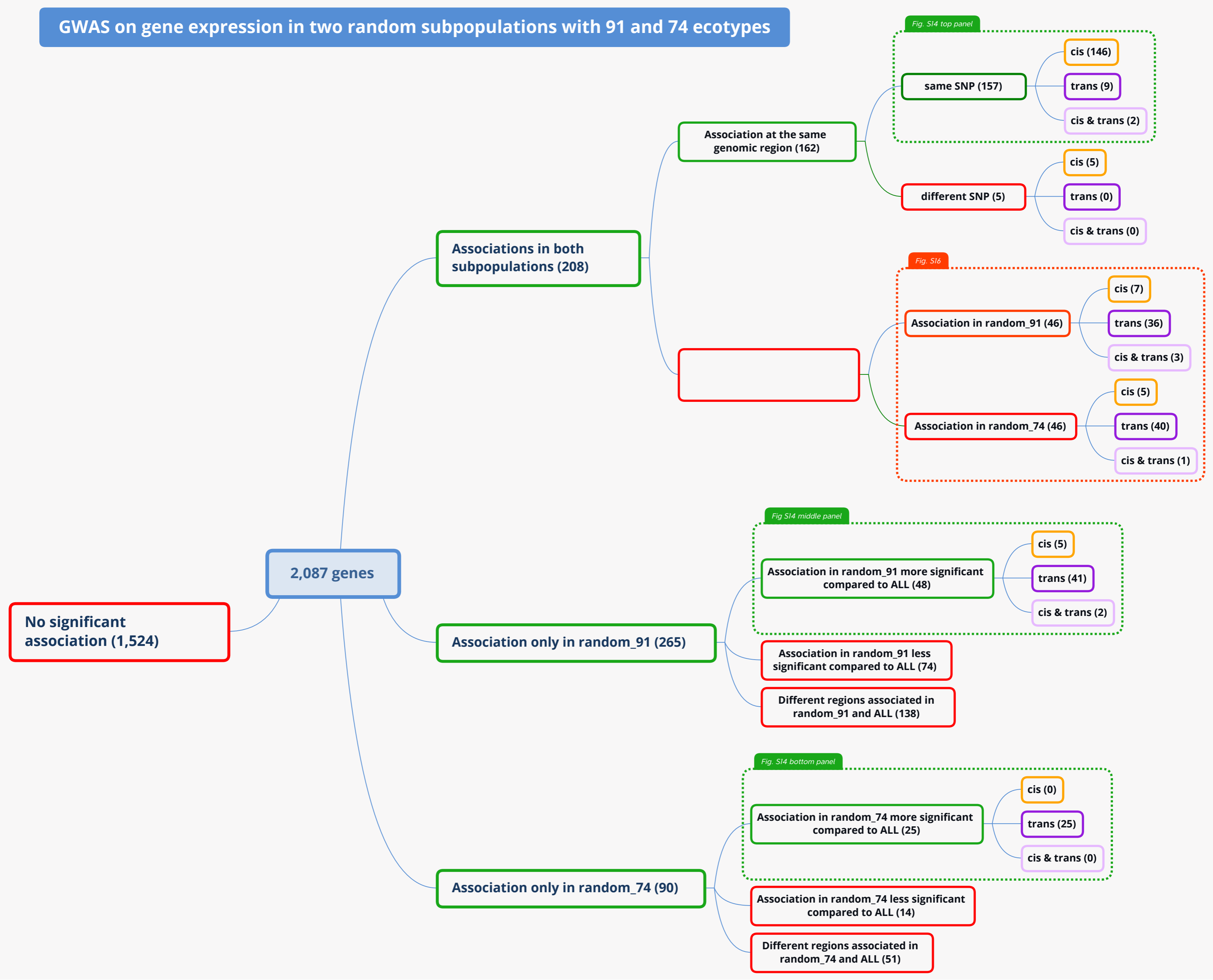

Supplement: msab208_Supplementary_Data [file msab208_supplementary_data.zip › Supp_15.pdf]

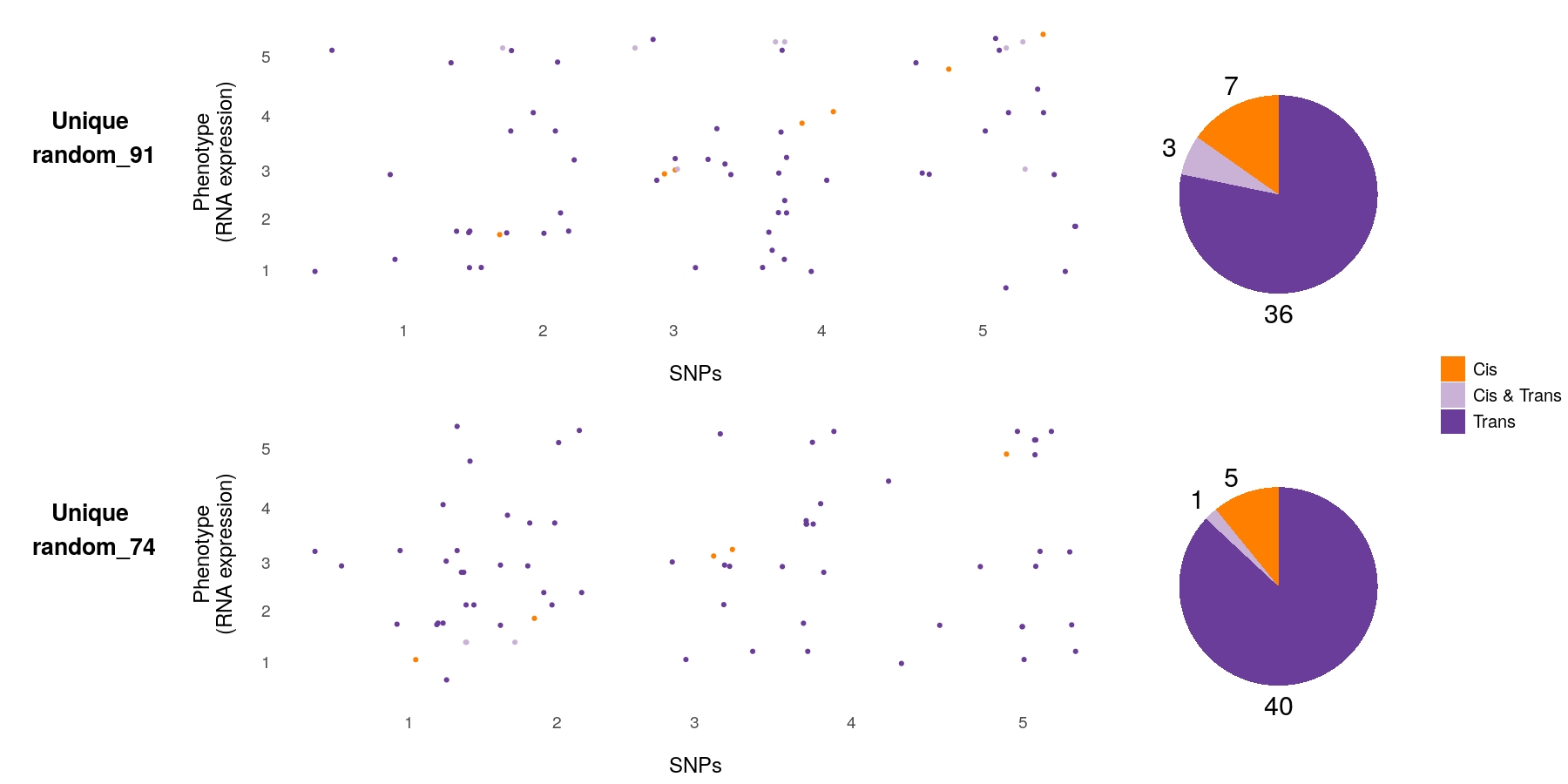

Supplement: msab208_Supplementary_Data [file msab208_supplementary_data.zip › Supp_16.jpeg]

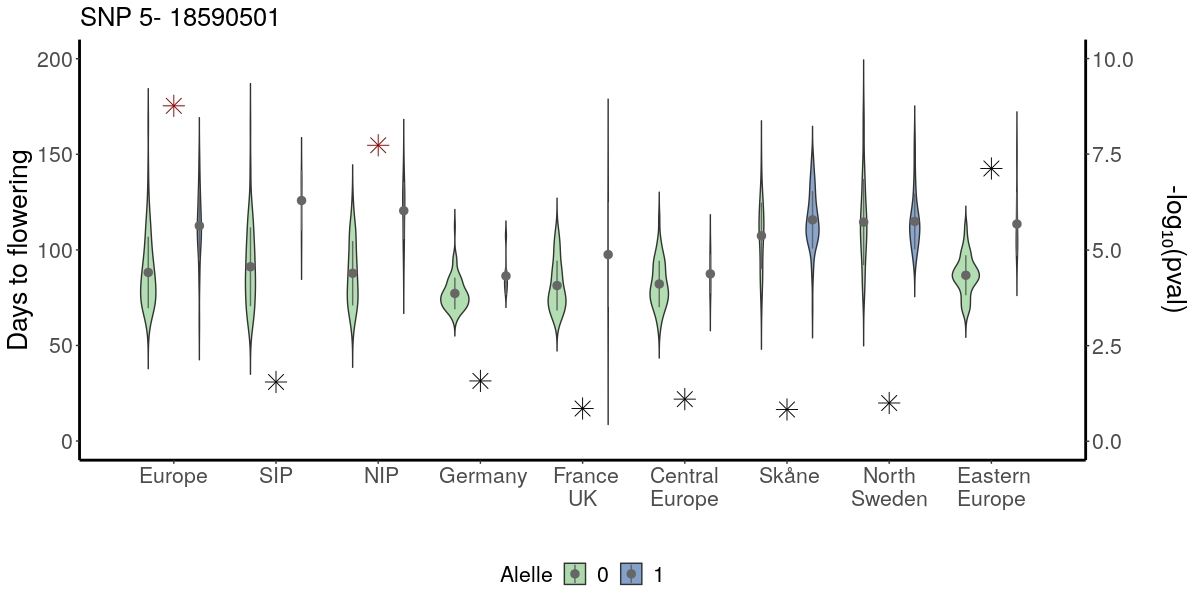

Supplement: msab208_Supplementary_Data [file msab208_supplementary_data.zip › Supp_2.jpeg]

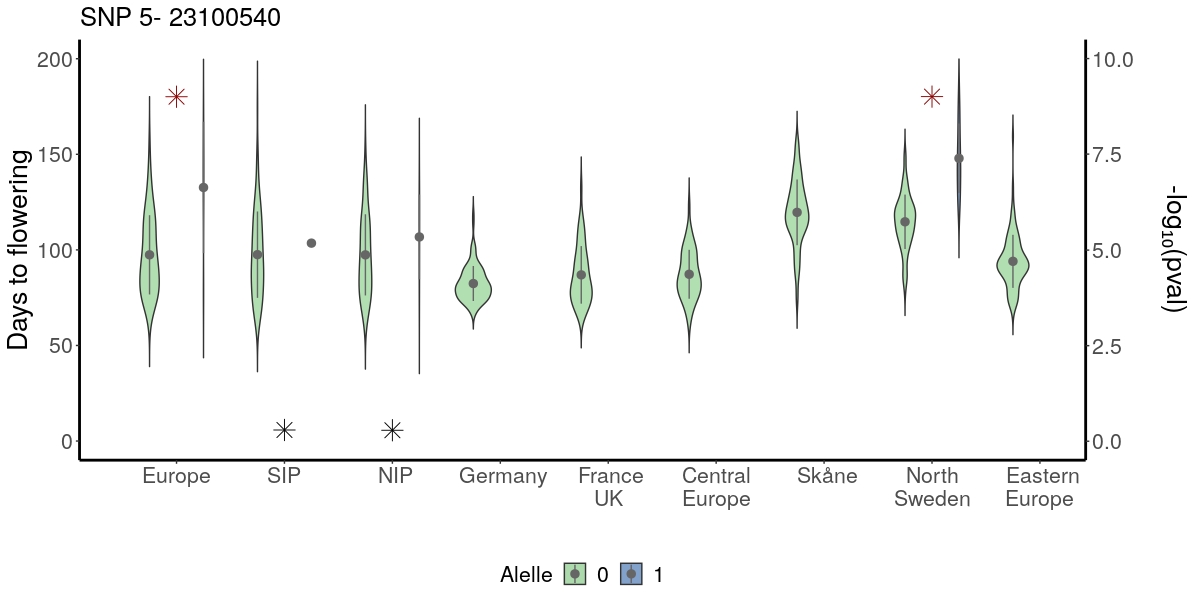

Supplement: msab208_Supplementary_Data [file msab208_supplementary_data.zip › Supp_3.jpeg]

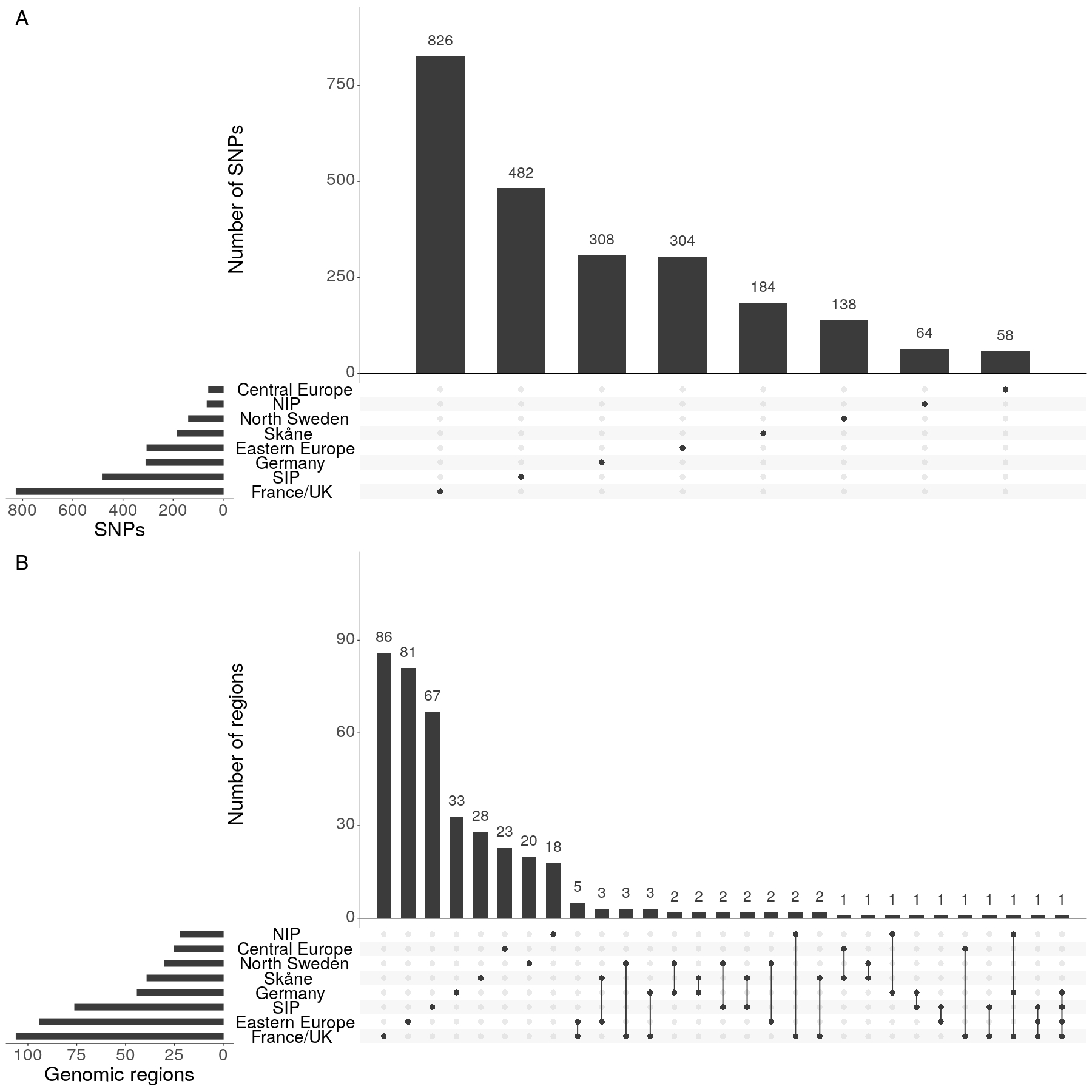

Supplement: msab208_Supplementary_Data [file msab208_supplementary_data.zip › Supp_4.jpeg]

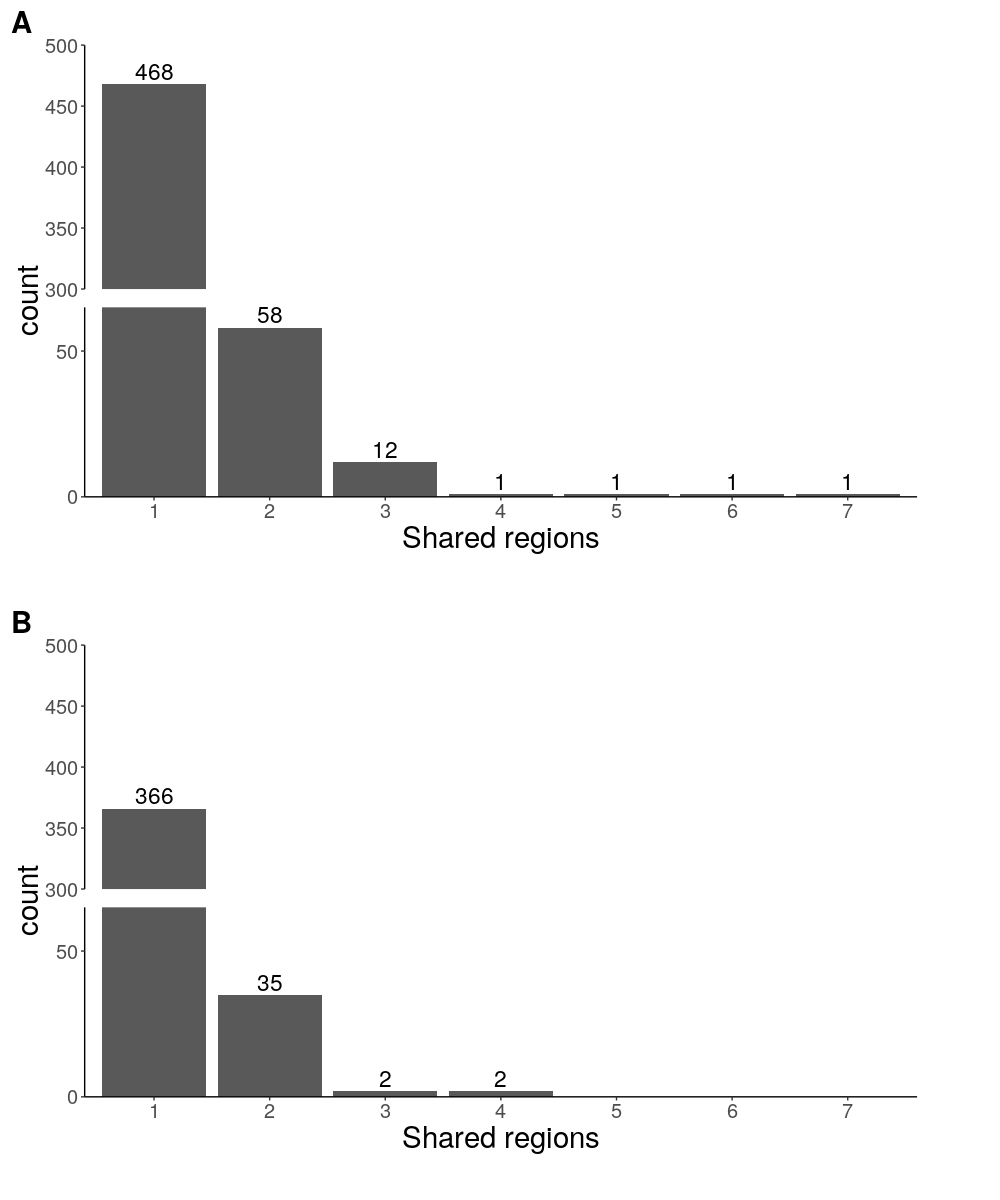

Supplement: msab208_Supplementary_Data [file msab208_supplementary_data.zip › Supp_5.jpeg]

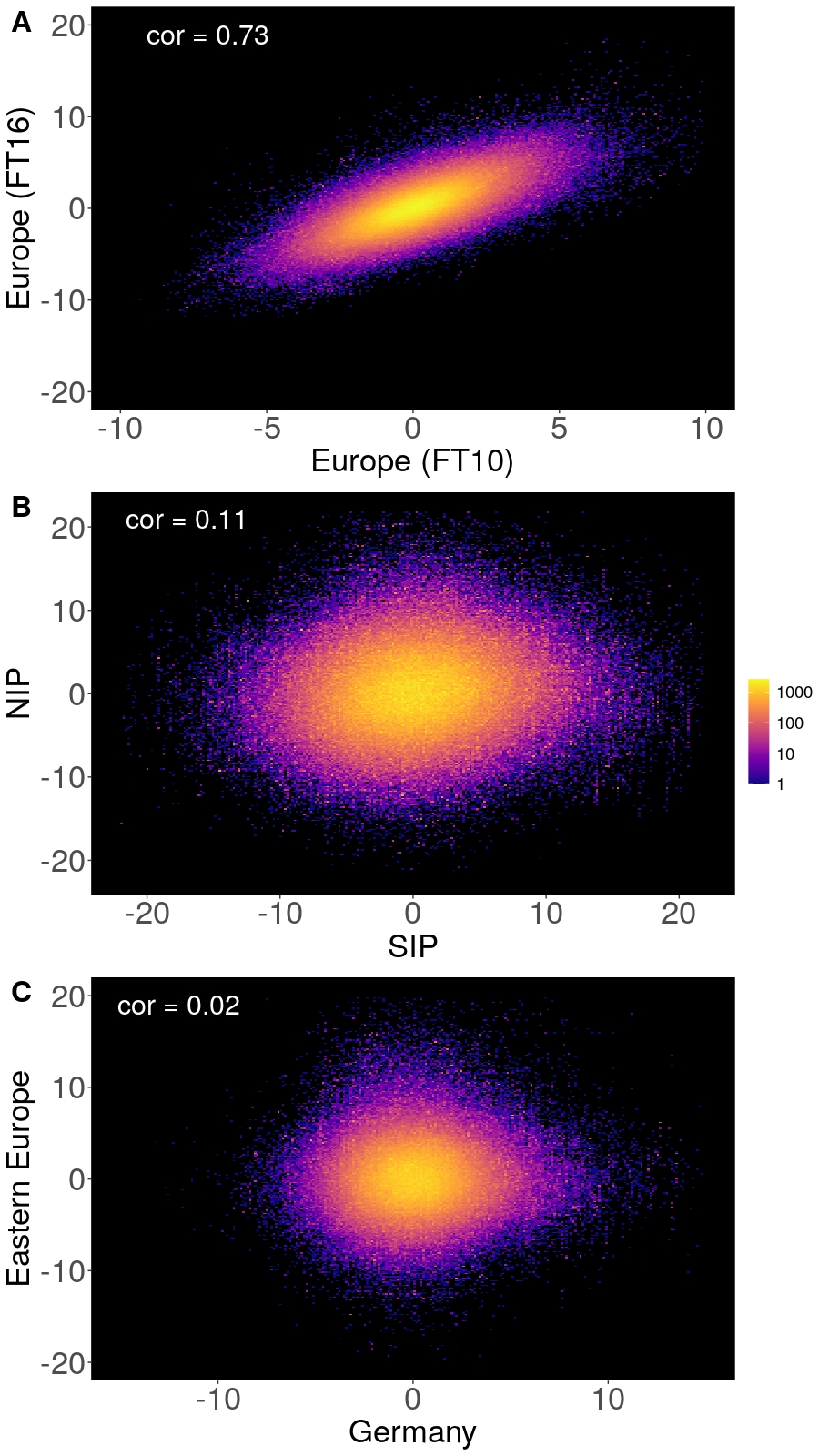

Supplement: msab208_Supplementary_Data [file msab208_supplementary_data.zip › Supp_6.jpeg]

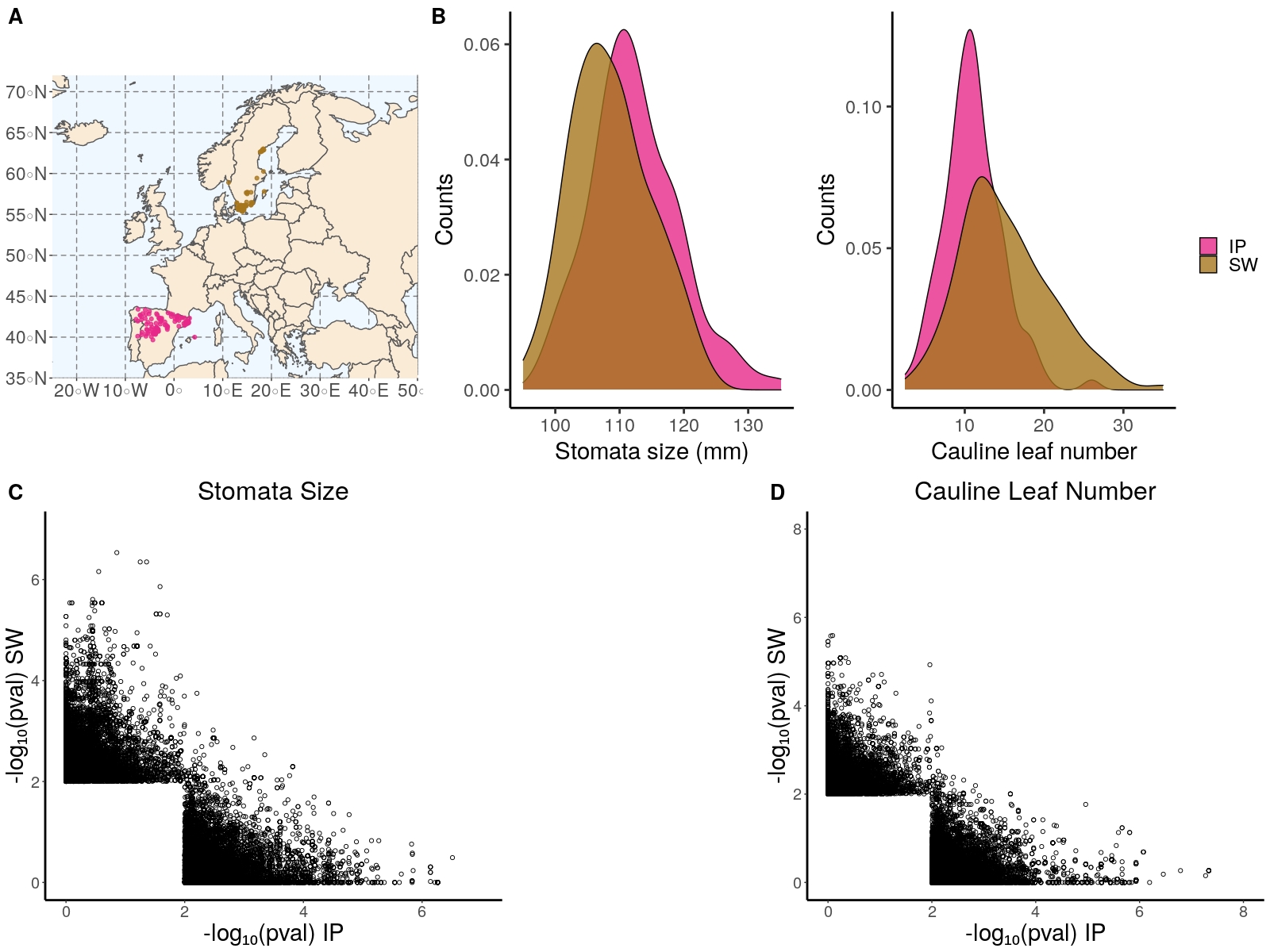

Supplement: msab208_Supplementary_Data [file msab208_supplementary_data.zip › Supp_7.jpeg]

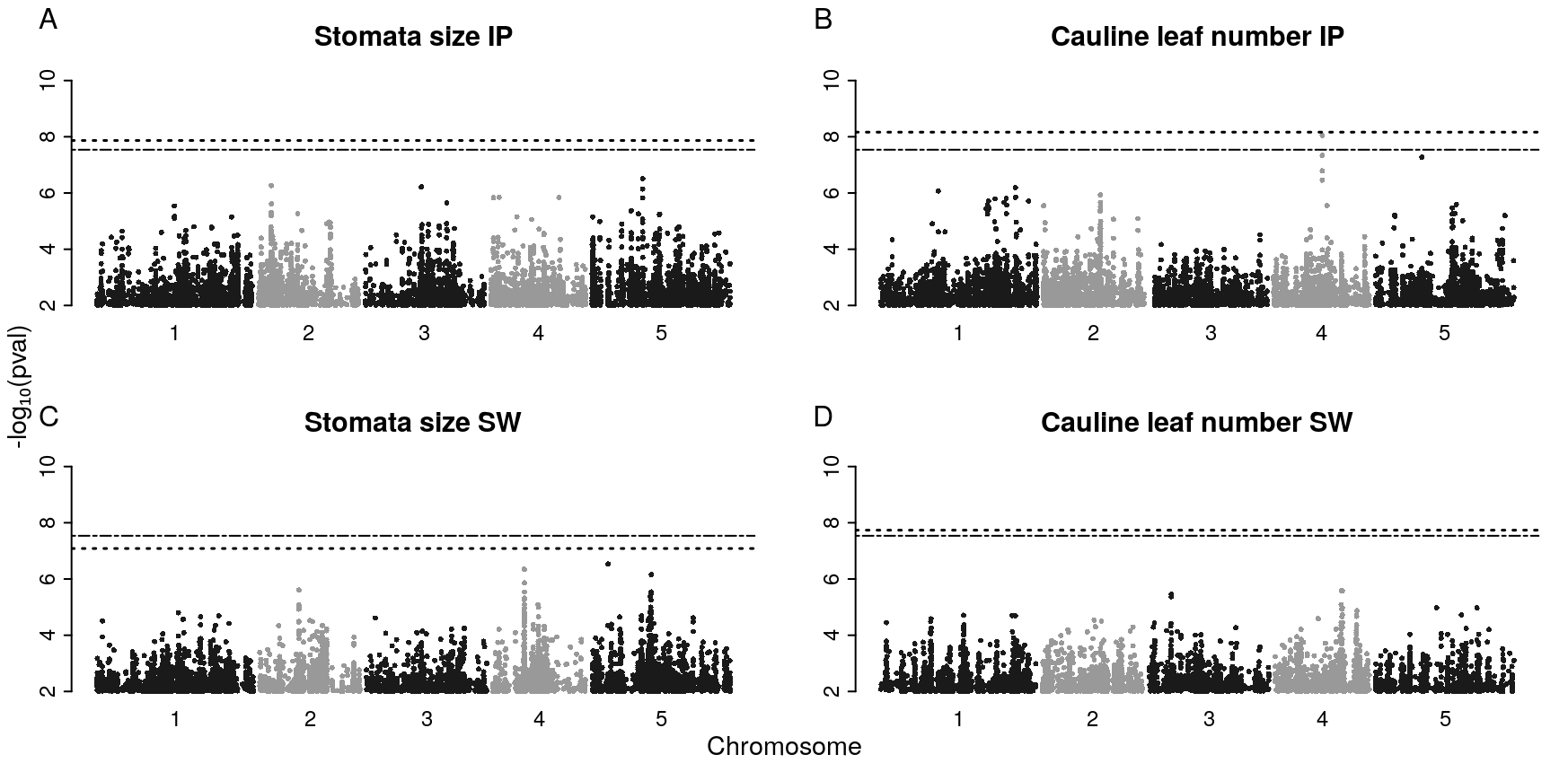

Supplement: msab208_Supplementary_Data [file msab208_supplementary_data.zip › Supp_8.jpeg]

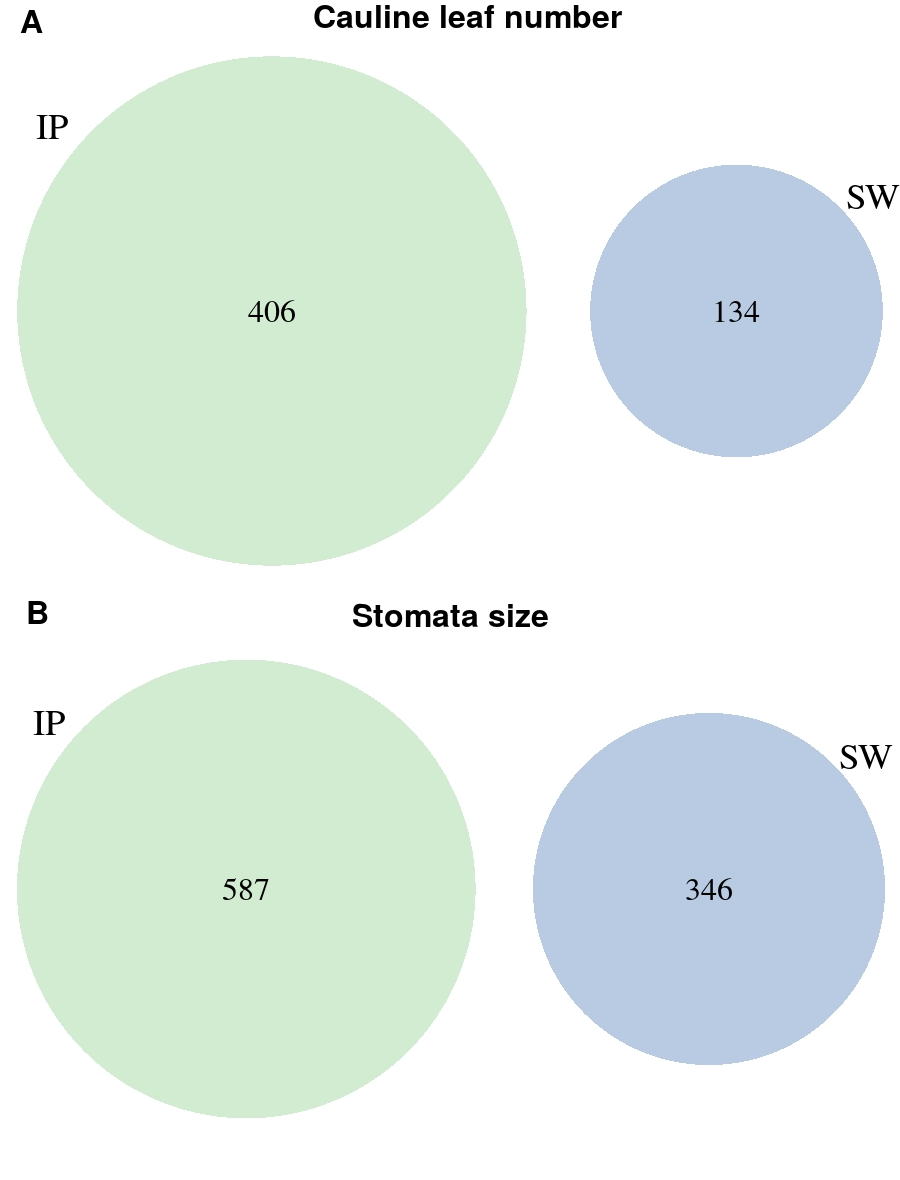

Supplement: msab208_Supplementary_Data [file msab208_supplementary_data.zip › Supp_9.jpeg]
